# Equity of Continuous Glucose Monitoring in Children and Young People With Type 1 Diabetes: A Systematic Review

**DOI:** 10.1155/pedi/8875203

**Published:** 2025-06-03

**Authors:** James Howard Dicks, Lucy Jane McCann, Abraham Tolley, Alice Barrell, Lucy Johnson, Isla Kuhn, John Ford

**Affiliations:** ^1^School of Clinical Medicine, University of Cambridge, Cambridge, UK; ^2^Wolfson Institute of Population Health, Queen Mary University of London, London, UK; ^3^School of the Biological and Biomedical Sciences, University of Cambridge, Cambridge, UK; ^4^University of Cambridge Medical Library, University of Cambridge, Cambridge, UK

## Abstract

**Background:** Socioeconomic status (SES) and ethnic inequalities in type 1 diabetes (T1D) outcomes are well-established. There is concern that unequal access to technologies, including continuous glucose monitoring (CGM), may increase disparities. This systematic review summarises the evidence for inequalities in the prevalence of CGM use for children and young people (CYP) and outcomes for CGM users.

**Methods:** Medline, Embase and Web of Science were searched for observational studies published between January 2000 and July 2023 which report CGM use stratified by any PROGRESS-Plus criteria for T1D patients under 26. Reports based in low- or middle-income countries, ≤500 participants or only reporting hybrid closed-loop systems were excluded. Primary outcomes were the proportion of patients using CGM and HbA1c of CGM users. Quality assessment was performed using the Newcastle–Ottawa Scale. Unadjusted odds ratios were calculated from the extracted summary data, though heterogeneity precluded meta-analysis. The protocol was preregistered with PROSPERO (CRD42023438139).

**Results:** Of the 3369 unique studies identified, 27 met the inclusion criteria. Thirty-three percent were of ‘good' or ‘very good' quality. We found decreased CGM use and higher discontinuation for low SES, low education, publicly insured and minority ethnic, especially Black, CYP. These associations were generally robust to adjustment for other sociodemographic variables, suggesting an independent effect. Lower SES inequalities were seen in countries where CGM is reimbursed. Although low SES and minority ethnicity were associated with poorer outcomes in general, for CGM users there was no significant association between domains of disadvantage and higher HbA1c, excepting parental education.

**Conclusions:** There are significant SES, ethnic and education inequalities in CGM use for CYP with T1D, particularly when reimbursement is limited. This inequity is contributing to inequalities in T1D outcomes. However, evidence suggests CYP benefit equally from CGM use, irrespective of ethnicity and SES. Increasing CGM funding and use is likely to reduce outcome inequalities.

## 1. Introduction

Continuous glucose monitoring (CGM) uses disposable subcutaneous sensors to measure interstitial glucose concentrations [1]. Data are transmitted to a smartphone either automatically at regular intervals (real-time CGM [rtCGM]) or when the sensor is manually scanned with a receiver device (intermittently scanned CGM [isCGM]) [2]. CGM data allow individuals and their healthcare professionals to optimise blood glucose control through timely and personalised adjustment of insulin doses [1]. CGM has been demonstrated to improve quality of life, time in range (TIR) and HbA1c of people living with diabetes in meta-analyses of randomised controlled trials (RCTs) [1] and real-world scenarios [3]. Additionally, CGM is cost-effective [4] and highly acceptable to patients [5]. Consequently, many countries are moving to fund CGM for type 1 diabetes (T1D) mellitus, particularly for children and young people (CYP), as good glycaemic control in early life reduces the risk of complications in adulthood [6, 7].

However, there is increasing concern that this technology may be contributing to health inequalities. Firstly, studies have suggested that disadvantaged groups have lower uptake of CGM [2], as has previously been demonstrated for insulin pumps [8]. Secondly, there is concern that even amongst CYP using CGM, it may differentially benefit more advantaged groups [9]. CGM, in particular isCGM, benefits individuals with the highest agency—those who scan and adjust their insulin doses most frequently [10]. Previous research has demonstrated that interventions that require a high degree of agency tend to increase inequalities [11].

This is a particularly pressing issue as inequalities in glycaemic control and diabetes complications for CYP from minority ethnic and socioeconomically disadvantaged backgrounds are already widespread, and UK data suggest they have been increasing over recent years [12]. Increasing access to diabetes technologies for CYP is one of NHS England's Core20PLUS5 priorities to reduce health inequalities [13]; characterising patterns of CGM use and benefit is vital to achieve this goal. However, inequalities in prevalence of CGM use have not yet been systematically evaluated and, despite suggestions that high agency interventions tend to disproportionately benefit the most advantaged groups, evidence is lacking as to whether this is the case for CGM.

In this review, demographic groups of interest are defined according to the Cochrane PROGRESS-Plus framework, an acronym used to describe several characteristics which stratify health opportunities and outcomes, encompassing place of residence, race/ethnicity/culture, occupation, gender, religion, education, socioeconomic status (SES) and social capital [14].

In summary, this systematic review seeks to explore the following:

1. The prevalence of CGM use and discontinuation rates across PROGRESS-Plus groups amongst CYP with T1D.
2. How glycaemic control and management varies across PROGRESS-Plus groups amongst CYP with T1D using CGM.

## 2. Materials and Methods

### 2.1. Search Strategy and Selection Criteria

We undertook a systematic review of observational studies that reported the use of CGM or outcomes for CGM users by PROGRESS-Plus groups in accordance with the Preferred Reporting Items for Systematic Reviews and Meta-Analyses (PRISMA) guidelines [15]. CGM was defined as either isCGM or rtCGM use.

Primary outcomes of interest were the proportion of participants using CGM and mean HbA1c of CGM users. Only studies reporting primary outcomes stratified by one or more PROGRESS-Plus group were included. Absolute numbers in each group, mean HbA1c of whole group, odds ratios, effect size, slope index of inequality and results of relevant statistical analyses were recorded. Additional outcomes of interest were CGM initiation and discontinuation rates, rates of hypoglycaemia and diabetic ketoacidosis, TIR and frequency of CGM use. All outcomes were prespecified in the PROSPERO protocol (CRD42023438139).

The search strategy was devised by an experienced information scientist (IK). Health inequalities and CGM search terms were adapted, respectively, from Prady et al. [16] and Elbalshy et al. [1]. Full details of the search strategy are outlined in (Supporting Information 1). Ovid Medline, Ovid Embase and Web of Science were searched on July 4, 2023. A grey literature search using the terms 'continuous glucose monitoring children inequalities' was undertaken on August 25, 2023, and the first 100 results were screened.

Inclusion criteria were (1) reports CGM use or HbA1c of CGM users stratified by any PROGRESS-Plus group; (2) reports outcomes for patients ≤25; (3) reports outcomes for patients with T1D; (4) observational studies; and (5) primary, secondary or tertiary healthcare setting.

Exclusion criteria were (1) reviews, letters, editorials and RCTs; (2) ≤500 participants; (3) studies published before January 1, 2000, as CGM was not commercially available until 1999 [1]; (4) based in low- and middle-income countries; (5) assessed hybrid closed-loop systems only; (6) assessed pregnant populations; and (7) only assessed interregional disparities.

Following initial removal of duplicates, all titles and abstracts were screened by JHD using Rayyan. Twenty percent of articles were randomly selected for independent screening by LJM to assess for systematic errors. Discrepancies were resolved through discussion with JF. Full texts were assessed against eligibility criteria by JHD, and all uncertainties resolved through discussion with LJM and JF. Reasons for exclusion were recorded (Supporting Information 2). Backwards and forwards citation searching of all included studies was performed.

Data were extracted by JHD using a predesigned standardised template (Supporting Information 3). A narrative synthesis was undertaken, grouping outcomes by PROGRESS-Plus criteria reported.

### 2.2. Data Analysis

Quality assessment for primary outcomes was performed independently by AT and AB. The Newcastle–Ottawa Scale was used for cohort studies [17], and an adapted version was used for cross-sectional studies [18]. Discrepancies were resolved through discussion with JHD.

All figures and odds ratios were generated using RStudio (2023.06.0+421). Given significant heterogeneity in populations and study periods, no meta-analytic synthesis was performed. Where given in Diabetes Control and Complications Trial (DCCT) units, all HbA1c values were converted to mmol/mol for ease of comparison.

## 3. Results

**Figure 1:**
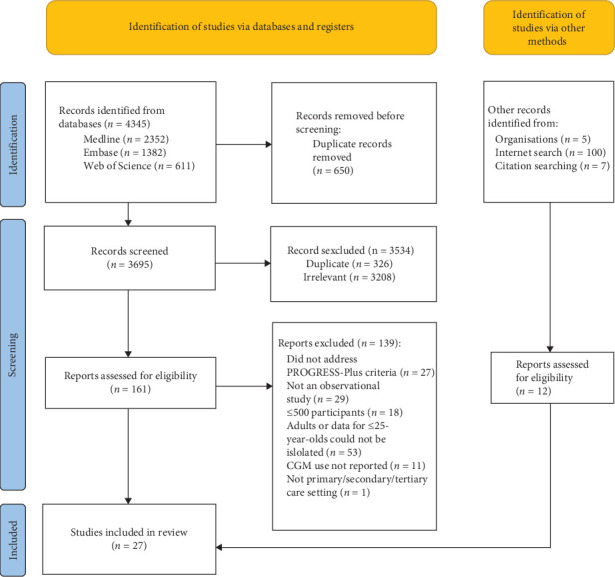
PRISMA 2020 flow diagram [14].

The search strategy identified 4345 results, of which 3369 were unique. The PRISMA flow diagram is shown in Figure 1. Study characteristics are summarised in Table 1. Twenty seven reports met inclusion criteria—21 journal articles, five annual audit reports and one master's thesis. The most common settings were the United States (*n* = 11), the United Kingdom (*n* = 5) and Germany (*n* = 3). The remainder included data from both the United States and Germany (*n* = 2); Canada (*n* = 2), New Zealand (*n* = 1) or other mainland European countries (*n* = 3). 56% (*n* = 15) were large multicentre studies. All studies reported CGM use and three studies additionally reported HbA1c outcomes for CGM users stratified by PROGRESS-Plus groups [2, 28, 35]. Regarding CGM use, 52% (*n* = 14) were rated as ‘moderate' and 33% (*n* = 9) ‘good' or ‘very good' quality (Supporting Information 4).

**Figure 2:**
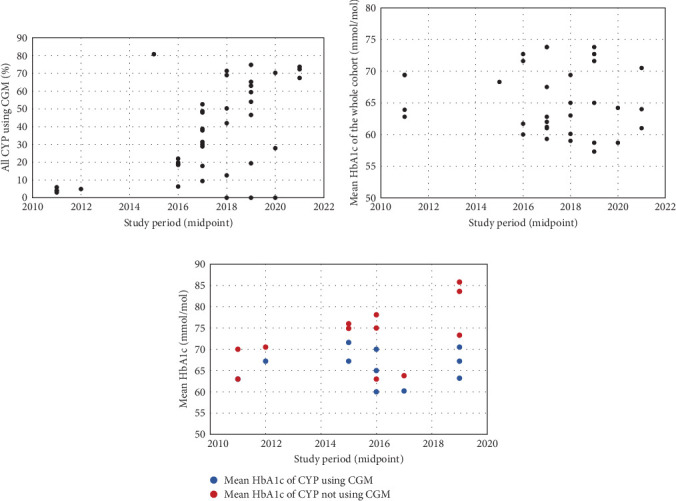
Trends in CGM use and HbA1c for children and young people: (a) CGM use, (b) mean HbA1c and (c) mean HbA1c stratified by CGM use. For studies conducted over multiple years, the midpoint of the study period was used.

The proportion of CYP using CGM ranged from 3.0% to 80.8% [21, 37], with a general increase over time (Figure 2a). Mean HbA1c, regardless of CGM use, ranged from 73.8 mmol/mol to 58.7 mmol/mol [19, 25, 28]. There were no clear temporal trends in outcomes (Figure 2b), though European children generally had better glycaemic control than their US counterparts. CGM users had lower HbA1c than nonusers in all but one study that reported these outcomes (*n* = 9), with the difference ranging from 0.0 to 16.4 mmol/mol [21, 23, 24, 26, 28, 32, 36–38]. This difference has generally increased over time, primarily due to poorer outcomes for CGM nonusers (Figure 2c).

### 3.1. Ethnicity

**Figure 3:**
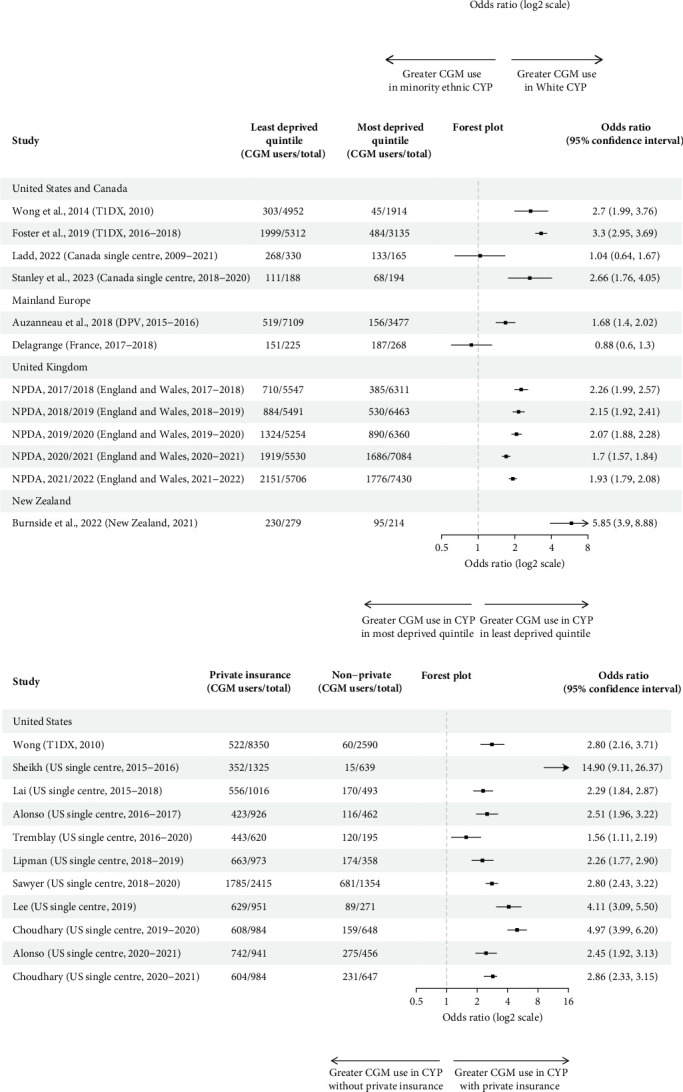
Forest plots of unadjusted odds ratios for CGM use. (a) Forest plot of unadjusted odds ratios for CGM use (white vs. minority ethnic CYP). (b) Forest plot of unadjusted odds ratios for CGM use (least deprived quintile vs. most deprived quintile CYP). (c) Forest plot of unadjusted odds ratios for CGM use (private insurance vs. no private insurance CYP). (d) Forest plot of unadjusted odds ratios for CGM use (male vs. female CYP).

Ten multicentre and nine single-centre studies reported CGM use by ethnicity (Table 2). Most were US-based (*n* = 13) or UK-based (*n* = 5). Seventy-nine percent(*n* = 15) found higher CGM usage for White CYP relative to all other ethnic groups, though only nine studies assessed the statistical significance of ethnic differences in CGM use. Seven studies performed unadjusted analysis, of which four demonstrated significantly higher CGM use in White, relative to Black and Hispanic, CYP [24, 29, 30, 32]. Of the remainder, one found significantly higher rates of CGM use for White children ≤13, but not for older CYP [23]; one found higher CGM use in White relative to Hispanic (*p* < 0.0001), but not Black (*p*=0.07) CYP [26]; and one found ethnicity was not significantly associated with CGM use [27]. Calculation of unadjusted odds ratios demonstrated White CYP had significantly higher odds of using CGM in all but one study (Figure 3a) [27]. Unadjusted odds ratios for not using CGM relative to White CYP ranged from 1.41 (1.21, 1.65) to 5.77 (3.69, 9.55) for Black CYP (*n* = 15) [12, 32], 1.52 (1.12, 2.13) to 4.10 (2.92, 5.90) for Hispanic CYP (*n* = 9) [ 23, 32] and 0.50 (0.10, 2.76) to 2.18 (1.82, 2.63) for Asian CYP (*n* = 7) (Supporting Information 5) [26, 44]. Black CYP had higher unadjusted odds of not using CGM than Hispanic patients in eight of the 10 studies that reported CGM usage of both groups (Supporting Information 5). All five studies that adjusted for key sociodemographic variables on multivariable analysis, including SES measures, demonstrated a significant association between CGM use and White ethnicity [2, 19, 29, 30, 32].

Three studies addressed additional facets of CGM use. One US single-centre study found that, whilst CYP of all ethnicities achieved relatively high CGM use, Black and Hispanic patients took significantly longer to start CGM, had fewer average days of use and were more likely to discontinue CGM relative to White patients [27]. Two further US-based studies demonstrated higher discontinuation rates for Black—but not Hispanic—CYP [30] and significantly lower rates of sensor wear for minority ethnic CYP [26].

White CYP were generally the group with the lowest HbA1c (*n* = 12), and Black CYP were the group with the highest (*n* = 8). These differences were statistically significant on unadjusted (*n* = 7) [ 2, 19, 22, 28, 29, 31, 32] and multivariate analysis (*n* = 5) [2, 19, 22, 31, 32]. Three studies reported outcomes for CGM users of different ethnicities (Table 2) [2, 28, 31]. One US study found that minority ethnicity was a significant predictor of higher HbA1c for CGM nonusers, but, for CGM users, ethnicity was not significantly associated with HbA1c [28]. For CYP in New Zealand, there was similarly no significant association between ethnicity and outcomes for CYP using rtCGM on multivariable modelling, but non-European ethnicity was associated with higher HbA1c for CGM nonusers and isCGM users [2]. One study found White ethnicity was associated with significantly greater TIR [31], and one found TIR did not significantly differ between ethnic groups [28].

### 3.2. Socioeconomic Status and Insurance Status

**Figure 4:**
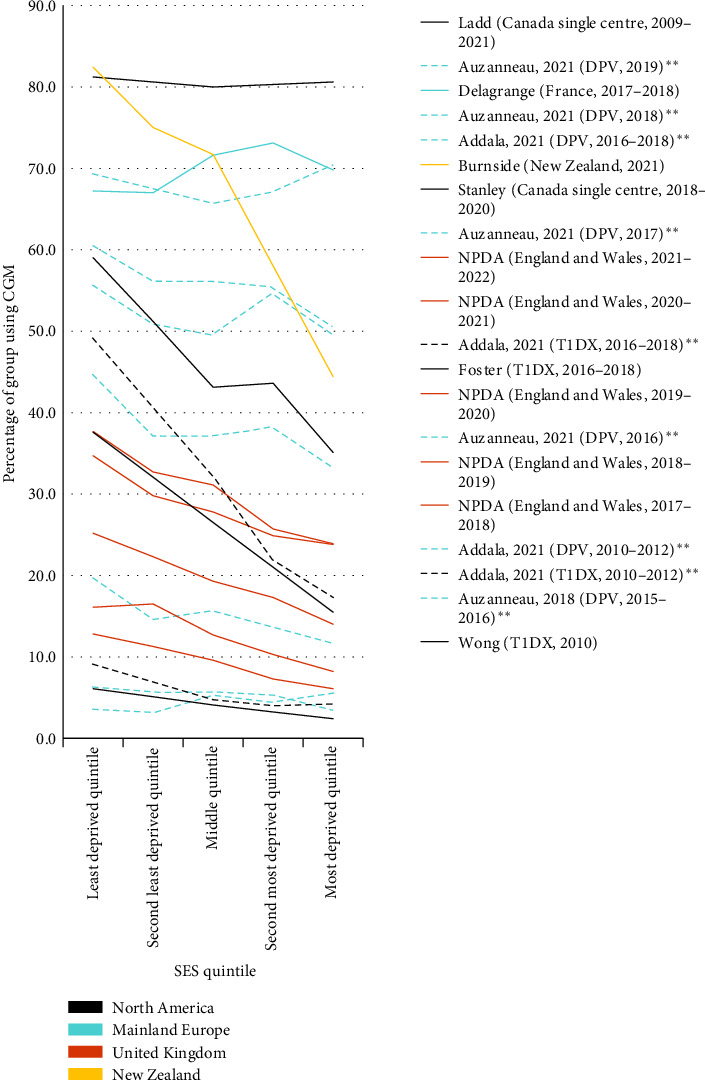
CGM use by socioeconomic quintile. *⁣*^*∗∗*^Data from Auzanneau et al. [34] and Auzanneau et al. [33] are taken from linear regression models adjusted for sex, age group, migration, diabetes duration and federal state. Data from Addala et al. [19] are taken from linear regression models adjusted for period, sex, age, diabetes duration, minority status, income/education/insurance by period interaction and income/education/insurance by minority status interaction. All other studies represented in this figure use unadjusted data.

Twelve multicentre and two single-centre studies reported CGM use by SES, quantified by the Index of Multiple Deprivation (*n* = 12) or household income (*n* = 2) (Table 3, Figure 4). Ten US studies further reported CGM use for CYP with public and private insurance.

Of the four studies that performed unadjusted analysis, three found significantly lower unadjusted CGM use in the most deprived quintile [2, 23, 36], and one Canadian study—in which CGM use was over 80% for all SES groups—found no significant difference [37]. Significantly greater unadjusted odds of using CGM for the least deprived group were demonstrated in all but two studies [37, 40], with a range of 0.88 (0.60, 1.30) to 5.85 (3.90, 8.88) (Figure 3b). Five studies performed analysis adjusted for other sociodemographic variables including ethnicity [2, 19, 33, 34, 37], of which four identified SES as a significant predictor of CGM use [2, 19, 33, 34].

Privately insured CYP had significantly higher unadjusted odds of using CGM for all studies for which calculation was possible, ranging from 1.56 (1.11, 2.19) to 14.90 (9.11, 26.37) (Figure 3c) [27, 32]. All studies that performed unadjusted (*n* = 5) [23, 24, 27, 30, 32] and adjusted (*n* = 3) [19, 30, 32] analysis found privately insured CYP were significantly more likely to use CGM. Publicly insured CYP also took longer to initiate CGM and used it less [27]. One study found those with public insurance were more likely to discontinue CGM [27], and one study found no significant differences in discontinuation rates [30].

HbA1c was greater in the most deprived than in the least deprived SES quintile in all studies in which it was assessed (*n* = 11). This difference was significant on multivariable (*n* = 4) [2, 19, 33, 40] and unadjusted (*n* = 3) [2, 36, 40] analyses in all but one study [37]. Private insurance was likewise significantly associated with lower HbA1c on adjusted analysis (*n* = 4) [19, 28, 31, 32]. Only one study performed subgroup analysis of CGM users, which showed no significant difference in outcomes for government and commercially insured children (Table 3) [28]. No studies performed subgroup analysis for household income or IMD.

### 3.3. Sex

Sixteen studies reported CGM use by sex (Table 4). Most demonstrated slightly higher CGM use for females, though only two of the 10 studies in which statistical analysis was performed found this difference to be significant [37, 39]. Unadjusted odds ratios demonstrated no significant association between sex and CGM use in 10 of the 14 studies where calculation was possible (Figure 3d). Females had significantly greater unadjusted odds of using CGM in three studies [23, 27, 29] and males in one [25]. Sex was not associated with discontinuation rates or days of use [27, 30].

Six studies reported HbA1c values by sex, of which five performed multivariable analysis—in none was sex significantly associated with HbA1c or TIR [2, 28, 31, 32, 35]. There was no significant difference in outcomes for male and female CYP using CGM [28, 35, 37].

### 3.4. Other PROGRESS-Plus Criteria

Three US studies assessed how CGM use varied according to parental education; lower parental education was significantly associated with lower CGM use on unadjusted and multivariable analysis [19, 23]. Other relevant criteria assessed included primary language (*n* = 3), migration background (*n* = 1), rural versus urban (*n* = 1), household structure (*n* = 1) and parental occupation (*n* = 1) (Table 5). Having parents who did not speak English [24, 27, 32], a migration background [34], single parent household [27] or parents who work in service/trade were all significantly associated with lower CGM use [27]. CGM use did not differ between rural and urban youth [24].

Lower parental education was a significant predictor of poorer glycaemic outcomes on multivariable analysis [19, 28]. Notably, in contrast to insurance status and ethnicity, low parental education remained significantly associated with high HbA1c in subgroup analysis of CGM users [28]. One study reported a significant association between English-speaking parents and lower HbA1c [32]. HbA1c was not reported by studies assessing migration background, rural versus urban, household structure or parental occupation.

In general, significant associations between domains of disadvantage and higher HbA1c remained significant after adjusting for CGM and pump use on multivariable modelling. More specifically, one Canadian study found rtCGM use—but not isCGM—to be a significant partial mediator of the relationship between SES deprivation and HbA1c, accounting for 12% of the difference [36].

## 4. Discussion

We found widespread inequalities in CGM use for CYP with T1D. Low SES, public insurance, low parental education and minority ethnicity—particularly Black ethnicity—were all consistently associated with lower CGM use. This is in keeping with data from adults and other diabetes technology, such as insulin pumps [8, 45]. Prevalence of CGM use was lower in low SES and minority ethnic groups, and these CYP used it less frequently, had less TIR and were more likely to discontinue its use. These associations were generally robust to adjustment for other key sociodemographic variables, suggesting that each domain of disadvantage had an independent effect.

Cost has been identified as a key barrier to CGM uptake in both quantitative and qualitative studies [19, 46], and fully subsidising CGM has been shown to dramatically increase uptake and decrease SES inequalities [47, 48]. This review supports the importance of CGM subsidies. In general, we identified steeper SES inequalities in countries with no reimbursement or commercial insurance systems [2, 19, 22]. Ethnic and SES inequalities were steepest in New Zealand, where CGM is not reimbursed through either public or private health insurance (Figure 4) [2]. In the United States, rtCGM is funded by commercial insurance following certification of medical need, though public Medicaid coverage varies by state [6]. As of 2022, 40 states provide CGM for CYP with T1D through Medicaid coverage, though before 2017 there was no recommendation for funding [20]. Inequalities identified by this review varied accordingly but were generally steeper than in Europe.

In Germany, reimbursement of rtCGM by statutory insurance, which covers ~90% of the population, started in summer 2016 [21]. This review found that, following an increase between 2010 and 2016, inequalities in CGM use reduced to the point where SES inequalities were no longer significant by 2019 on multiple regression modelling [19, 33, 34]. Similarly, in the United Kingdom, where CGM technology has been funded for all children with considerable fear or risk of hypoglycaemia since 2015 [7], both socioeconomic and ethnic inequalities decreased between 2018 and 2021, though increased again in 2022 [12]. However, it is of note that migration background inequalities were not completely eliminated by reimbursement in Germany [34], and UK data demonstrated persistent SES and ethnic inequalities in CGM use despite broad eligibility for fully-funded CGM [12].

Inequalities in clinician recommendation of CGM may contribute to disparities that persist despite CGM funding. White, high SES youth are more frequently offered diabetes technology [8], and provider gatekeeping has been identified as a key barrier to technology use in minority youth [49]. This may be because some common criteria for eligibility, such as performing a minimum number of daily blood glucose checks [6], favour high SES, White CYP [3]. Other criteria, such as fear and risk of hypoglycaemia [6, 7], may also more frequently apply to these groups. As this review has consistently demonstrated, less disadvantaged CYP tend to have better glycaemic control, which has been associated with greater risk of hypoglycaemia [50]. Only one study identified by this review reported rates of hypoglycaemia for different demographic groups; they identified significantly higher rates of hypoglycaemia in CYP from areas with lower Indices of multiple deprivation, in keeping with this hypothesis [33]. However, evidence is mixed, as hypoglycaemia has also been associated with minority ethnicity and low SES elsewhere [ 50, 51]. Another possible contributor to inequalities is that prescribing guidelines may not be consistently followed. In the United States, which comprised the majority of studies identified by this review, only a minority of providers reported using consensus guidelines when prescribing insulin pumps; most relied on subjective factors such as patient motivation, lifestyle factors and perceived ability to benefit [52, 53]. It has been suggested that implicit bias may exacerbate ethnic and SES inequalities in CGM use through similar mechanisms [54].

Variation in acceptability to different demographic groups may further contribute to differences in CGM uptake. Research has indicated concerns amongst minority parents and youth that conspicuous technology may increase stigma, due to poor understanding of T1D in their communities [55, 56]. Lack of confidence in using technology and higher levels of alarm fatigue for CYP with poor glycaemic control have also been identified as barriers to CGM use [55, 57]. These factors may contribute to lower uptake and further may explain lower usage rates and higher rates of discontinuation observed in this review for minority ethnic, low SES CYP.

Though the majority of research has focused on CGM uptake, concerns have been raised that disadvantaged youth may derive less benefit from CGM [9]. Firstly, CGM yields the greatest benefit when used concurrently with insulin pumps, especially when these are combined in hybrid closed-loop systems [58]. Inequalities in pump access are widespread [8], and so increasing CGM use according to similar patterns may be expected to compound existing disparities. Secondly, CGM, especially isCGM, is a high-effort intervention, requiring frequent adjustment of diet, exercise and insulin doses in light of blood glucose information [1]. Those who use CGM and adjust doses more frequently have been shown to derive greater benefit [10, 59], as do patients with higher level of health numeracy [60]. As lower diabetes numeracy and reduced frequency of blood glucose monitoring are associated with minority ethnicity, low SES and low parental education [61, 62], it may be expected that disadvantaged groups would derive less benefit from CGM.

However, we found similar improvements in glycaemic control for all ethnicities and insurance groups using CGM, though evidence was limited [2, 28]. This concurs with previous data from adults [63], though may be somewhat confounded by the fact that CGM leads to more drastic change for those with higher initial HbA1c [10]. In contrast, low parental education was associated with poorer outcomes on multivariate analysis, even for CGM users, though data were limited [28]. This may implicate health numeracy as a continued source of outcome inequality, even if access inequalities were completely eliminated.

### 4.1. Strengths and Limitations

This is the first systematic review to examine inequalities in CGM use and outcomes for CYP internationally. We compared data through calculation of unadjusted odds ratios where possible, though data were not pooled due to heterogeneity across studies. We examined multiple domains of disadvantage and assessed socioeconomic inequalities through income, education and insurance; however, there was insufficient data to assess multiple disadvantage.

Many studies did not perform multivariable analysis, so some results may be subject to confounding. There is also a risk of publication bias, either due to reluctance of healthcare organisations to publish findings which may suggest increasing inequalities or researchers focusing on highlighting disparities. The search terms used in this review may have overrepresented the latter. However, we have included a range of grey literature from established national datasets to mitigate this potential source of bias. Finally, we could not robustly assess differential outcomes for isCGM and rtCGM use due to insufficient reporting.

## 5. Conclusions

### 5.1. Implications for Policy and Future Research

Increased data sharing is paramount to allow for equity monitoring of data and equity-focused quality improvement in CGM use. The recent change to UK guidelines recommending CGM for all CYP provides a key opportunity to assess the impact of removing all CGM access restrictions on inequalities [7]. We found that full reimbursement for CGM is likely to be effective in reducing inequalities in access, especially those relating to SES, and that most disadvantaged groups benefit equally from CGM use. Where universal provision is unfeasible, subjective or inequitable eligibility criteria, such as glucose monitoring frequency, should be removed and clear guidelines published. Evidence-based interventions to reduce inequity in prescribing, such as insurance navigation and social needs assessments, may also be of benefit [64].

We found that lower parental education was associated with outcome inequality amongst CGM users. Increasing access to technologies that reduce the burden on patients and families to understand and act on health information, such as hybrid closed-loop systems or improved remote monitoring, may reduce these inequalities [65, 66].

Finally, this study found adjusting for CGM use in multivariable models did not eliminate HbA1c inequalities, suggesting CGM access is only one of several pathways by which the social determinants of health manifest with regard to diabetes care. This supports modelling studies showing that equalising diabetes treatment regimens would improve, but not eliminate, diabetes outcome disparities [67]. Continued research is required to identify further sources of inequality.

## Figures and Tables

**Table 1:** Study characteristics.

Study	Study type	Study setting	PROGRESS-Plus addressed	Population characteristics	Study period	Population size	Proportion using CGM	Mean HbA1c of cohort (mmol/mol)	Mean HbA1c of CGM users (mmol/mol)	Mean HbA1c of those not using CGM (mmol/mol)	CGM funding
Addala et al. [19]	Cross-sectional	United States (T1DX)	Ethnic minority status SES (household income) Insurance status Parental education	Age <18 years, clinical diagnosis of T1D, treated with insulin, T1D duration ≥1 year, recorded ethnicity and address/SES information	01/01/2010–31/12/2012	10,463	5.9% (rt and is)	69.4	NA	NA	Nonuniversal insurance system. rtCGM is funded by commercial insurance following provision of a certificate of medical need; Medicaid suggested coverage of CGM in 2017, but policy varies by state [20]
					01/01/2016–31/12/2018	9979	30.1% (rt and is)	73.8	NA	NA	
		Germany (DPV)	Ethnic minority status SES (IMD income deprivation) Parental education		01/01/2010–31/12/2012	23,167	4.0% (rt and is)	63.9	NA	NA	Statutory insurance (90% of population): rtCGM refunded since September 2016 if poor glycaemic control and/or severe hypoglycaemia. Private insurance (10%): depends on contract
					01/01/2016–31/12/2018	26,670	48.7% (rt and is)	62.8	NA	NA	

DeSalvo et al. [21]	Cross-sectional	United States (T1DX)	Ethnic minority status. Sex	Age <18 years, T1D duration ≥1 year	01/01/2011–31/12/2011	11,608	4% (rt and is)	69.4	63	70	Nonuniversal insurance system. rtCGM is funded by commercial insurance following provision of a certificate of medical need; Medicaid did not cover CGM for this time period [20]
					01/01/2016–31/12/2016	8186	19% (rt and is)	72.7	65	75	
		Germany (DPV)			01/01/2011–31/12/2011	17,399	3% (rt and is)	62.8	63	63	Statutory insurance (90% of population): rtCGM refunded since September 2016 if poor glycaemic control and/or severe hypoglycaemia. Private insurance (10%): depends on contract
					01/01/2016–31/12/2016	20,964	22% (rt and is)	61.7	60	63	

Foster et al. [22]	Cross-sectional	United States (T1DX)	Ethnicity SES (household income)	Age <26 years	01/01/2016–31/03/2018	10,099^a^	28.9% (type not stated)^a^	NA	NA	NA	Nonuniversal insurance system. rtCGM is funded by commercial insurance following provision of a certificate of medical need; Medicaid suggested coverage of CGM in 2017, but policy varies by state [20]

Wong et al. [23]	Cross-sectional	United States (T1DX)	Ethnicity Household income Insurance status Sex Parental education	Age <26, completed survey questions related to CGM device use 1 year after enrolment in the T1DX registry	T1DX registry commenced enrolment in September 2010. No end cut-off given	12,651^b^	4.9% (rt only)^b^	NA	67.2	70.5	Nonuniversal insurance system. rtCGM is funded by commercial insurance following provision of a certificate of medical need; Medicaid did not cover CGM for this time period [20]

Sawyer et al. [24]	Cross-sectional	United States (Barbara Davis Center for Diabetes, Colorado)	Ethnicity Sex Primary language Place of residence Insurance status	Age <22 years, T1D duration >3 months, resident of Colorado, available HbA1c results	01/01/2018–31/12/2020	4003	65.2% (rt and is)	72.7	65.0 (CGM + pump) 70.5 (CGM + MDI)	85.8	Nonuniversal insurance system. rtCGM is funded by commercial insurance following provision of a certificate of medical need. Colorado Medicaid fully funds CGM for all patients <22

Alonso et al. [25]	Cross-sectional	United States (Barbara Davis Center for Diabetes, Colorado)	Ethnicity Sex Insurance status	Age <22 years, T1D duration >3 months, available recorded HbA1c	01/10/2016–31/10/2017	2827	38.8% (type not stated)	73.8	NA	NA	Nonuniversal insurance system. rtCGM is funded by commercial insurance following provision of a certificate of medical need. Colorado Medicaid fully funds CGM for all patients <22
					01/10/2020–31/03/2021	2731	72.3% (type not stated)	70.5	NA	NA	

Ravi et al. [26]	Cohort	United States (Large tertiary academic practice in Aurora, Colorado)	Ethnicity Sex	Age cut-off not explicitly stated but based in a paediatric diabetes clinic, covered by Colorado Medicaid, first outpatient encounter within the study period	01/07/2015–31/07/2017	892	19.8% (type not stated)	NA	70	78.1	Nonuniversal insurance system. rtCGM is funded by commercial insurance following provision of a certificate of medical need. Colorado Medicaid provided CGM for all CYP <22 on provision of documentation of hypoglycaemia, either by meter download or oral report

Tremblay et al. [27]	Cohort	United States (Boston Children's Hospital)	Ethnicity Sex Primary language Insurance status Household structure Parental occupation	Age 2–25 years at diagnosis, followed for ≥1 year	01/012016–31/12/2020	815	69% (Dexcom [rt] only)	NA	NA	NA	Nonuniversal insurance system. rtCGM is funded by commercial insurance following provision of a certificate of medical need; Medicaid suggested coverage of CGM in 2017, but state-specific reimbursement policy not stated in text [20]
				Aged 2–25 years, meaningful Dexcom CGM use	17/01/2015–17/03/2021	1391	NA	69.4	NA	NA	

Lee et al. [28]	Cross-sectional	United States (C.S. Mott Children's Hospital, Michigan)	Ethnicity Sex Parental education Insurance status	Age cut-off not explicitly stated but based in a paediatric diabetes clinic. T1D duration >6 months, treated with insulin, available recorded HbA1c	01/01/2019–31/12/2019	1212	54% (rt and is)	73.8	67.2	83.6	Nonuniversal insurance system. rtCGM is funded by commercial insurance following provision of a certificate of medical need; Medicaid suggested coverage of CGM in 2017, but state-specific reimbursement policy not stated in text [20]

Lipman et al. [29]	Cross-sectional	United States (Children's Hospital of Philadelphia)	Ethnicity Insurance status	Age <18 years, T1D duration ≥2 years	01/10/2018–31/12/2019	1331	63% (type not stated)	NA	NA	NA	Nonuniversal insurance system. rtCGM is funded by commercial insurance following provision of a certificate of medical need; Medicaid suggested coverage of CGM in 2017, but state-specific reimbursement policy not stated in text [20]

Lai et al. [30]	Cross-sectional	United States (Children's Hospital of Philadelphia)	Ethnicity Sex Insurance status	Age <17 years, not using CGM at initiation of study period, address in Philadelphia	01/01/2015–31/12/2018	1509	48% (rt only)	NA	NA	NA	Nonuniversal insurance system. rtCGM is funded by commercial insurance following provision of a certificate of medical need; Medicaid suggested coverage of CGM in 2017, but state-specific reimbursement policy not stated in text [20]

Choudhary et al. [31]	Cross-sectional	United States (Children's Medical Centre Dallas)	Ethnicity Sex Insurance status	Age cut-off not explicitly stated but based in a paediatric diabetes clinic. ≥1 outpatient visit	15/3/2019–14/3/2020	1631	NA (rt and is)	NA	NA	NA	Nonuniversal insurance system. rtCGM is funded by commercial insurance following provision of a certificate of medical need; Texas Medicaid began approving reimbursement for CGM in April 2020
					15/03/2020–14/03/2021						

Sheikh et al. [32]	Cross-sectional	United States (Texas Children's Hospital)	Ethnicity Sex Primary language Insurance status	Age < 26 years, T1D duration ≥ 1 year, ≥1 clinic visit in preceding year	01/07/2015–30/06/2016	1992	18.5% (rt and is)	71.6	71.6	76	Nonuniversal insurance system. rtCGM is funded by commercial insurance following provision of a certificate of medical need; Medicaid did not cover CGM for this time period [6]

Auzanneau et al. [33]	Cross-sectional	Germany (DPV)	SES (IMD)	Age <20 years, German residence documented in the DPV	01/01/2015–31/12/2016	29,284	6.3% (rt and is)	60 (median)	NA	NA	Statutory insurance (90% of population): isCGM fully funded since July 2019. rtCGM refunded since September 2016 if poor glycaemic control and/or severe hypoglycaemia. Private insurance (10%): depends on contract

Auzanneau et al. [34]	Cross-sectional	Germany (DPV)	SES (IMD) Sex Migration background	Age <26 years, treated with insulin, T1D duration ≥3 months, ≥1 visit documented between 2016 and 2019	01/01/2017–31/12/2017	25,442	17.9% (rt and is)	59.3 (median)	NA	NA	Statutory insurance (90% of population): isCGM fully funded since July 2019. rtCGM refunded since September 2016 if poor glycaemic control and/or severe hypoglycaemia. Private insurance (10%): depends on contract
					01/01/2018–31/12/2018	25,807	42% (rt and is)	60.1 (median)	NA	NA	
					01/01/2019–31/12/2019	26,218	59.4% (rt and is)	58.7 (median)	NA	NA	
					01/01/2020–31/12/2020	26,628	70.3% (rt and is)	58.7 (median)	NA	NA	

Kordonouri et al. [35]	Cross-sectional	Germany (Children's Hospital Auf Der Bult, Hannover)	Sex	Age <22 years, ≥1 visit to the outpatient clinic	01/07/2017–30/06/2018	700	31.4% (6.4% rt, 25% is)	61	60	NA	Statutory insurance (90% of population): isCGM offered to all ≥4 years, rtCGM offered to those with hypoglycaemia risk, sensor-augmented CSII offered to those with high risk/history of severe hypoglycaemia

Stanley et al. [36]	Cross-sectional	Canada (Hospital for Sick Children diabetes clinic, Toronto)	SES (material deprivation)	Age <19 years, valid postcode	01/06/2018–31/05/2020	813	46.6% (20.8 rt, 23.2 is)	71.6	63.2 (rtCGM)	73.3	Universal healthcare. Ontario fully funded isCGM for selected patients as of September 2019

Ladd [37]	Cohort	Canada (single centre in Ottawa)	SES (economic dependency) Sex Other IMD metrics (residential instability, ethnocultural composition, situational vulnerability)	Age <18 years, residing in Ontario	01/04/2009–30/09/2021	595	80.8% (48.4% rt, 32.4% is)	68.3	66.1 (before starting CGM) 67.2 (after commencing CGM)	74.9	Universal healthcare. Ontario fully funded isCGM for selected patients as of September 2019

Bratke et al. [38]	Cross-sectional	Norway (Norwegian Childhood Diabetes Registry)	Sex	Age <19 years	01/01/2017–31/12/2017	2623	52.6% (rt and is)	62	60.2	63.8	Universal healthcare. CGM available to all children and adolescents with type 1 diabetes

Burnside et al. [2]	Cross-sectional	All regional diabetes centres in New Zealand	Ethnicity SES (IMD) Sex	Age <15 years, under a secondary care paediatric diabetes service in New Zealand	01/10/2021	1209	67.4% (27.2% rt, 40.2% is)	64 (median)	NA	NA	Universal healthcare. CGM systems are not funded by government-funded healthcare or private insurance providers

Šumník et al. [39]	Cross-sectional	Czechia (CENDA Registry of 49 paediatric diabetes centres)	Sex	Age <20 years	01/01/2017–31/12/2017	3130	37.9% (37.4% rt, 0.5% is)	61.2 (median)	NA	NA	Universal public insurance. CGM is mostly reimbursed for all children and adolescents: 0–7 years: 24 6-day sensors/year reimbursed fully, 37 reimbursed at 75% 8–18 years: 24 6-day sensors/year reimbursed fully, 19 reimbursed at 75%
					01/01/2018–31/12/2018	3211	50.3% (42.9% rt, 7.3% is)	59 (median)			
					01/01/2019–31/12/2019	3397	74.8% (40.7% rt, 34.1% is)	57.3 (median)			

Delagrange et al. [40]	Cross-sectional	France (7 paediatric diabetes specialist centres, SW France)	SES (European IMD)	‘Children', T1D duration ≥1 year	02/11/2017–03/05/2018	1154	71.4% (64.8% is, a3% sensor-augmented pump (rt))	63	NA	NA	Universal public insurance. Does not fund CGM [6]

NPDA, 2021/2022 [12]	Cross-sectional	NPDA (173 paediatric diabetes units England and Wales)	Ethnicity SES (IMD)	Age <24 years, under the care of a consultant paediatrician	01/04/2021–31/03/2022	31,349	73.7% (30% rt, 43.7% is)	61 (median)	NA	NA	Universal healthcare. CGM offered to all children at risk of or with significant fear of hypoglycaemia

NPDA, 2020/2021 [41]	Cross-sectional	NPDA (171 paediatric diabetes units England and Wales)	Ethnicity SES (IMD)	Age <24 years, under the care of a consultant paediatrician	01/04/2020–31/03/2021	29,892	27.9% (rt only)	64.2	NA	NA	Universal healthcare. CGM offered to all children at risk of or with significant fear of hypoglycaemia

NPDA, 2019/2020 [42]	Cross-sectional	NPDA (166 paediatric diabetes units England and Wales)	Ethnicity SES (IMD)	Age <24 years, under the care of a consultant paediatrician	01/04/2019–31/03/2020	27,653	19.4% (rt only)	65	NA	NA	Universal healthcare. CGM offered to all children at risk of or with significant fear of hypoglycaemia

NPDA, 2018/2019 [43]	Cross-sectional	NPDA (175 paediatric diabetes units England and Wales)	Ethnicity SES (IMD)	Age <24 years, under the care of a consultant paediatrician	01/04/2018–31/03/2019	28,597	12.6% (rt only)	65	NA	NA	Universal healthcare. CGM offered to all children at risk of or with significant fear of hypoglycaemia

NPDA, 2017/2018 [44]	Cross-sectional	NPDA (173 paediatric diabetes units in England and Wales)	Ethnicity SES (IMD)	Age <24 years, under the care of a consultant paediatrician	01/04/2017–31/03/2018	28,300	9.4% (rt only)	67.5	NA	NA	Universal healthcare. CGM offered to all children at risk of or with significant fear of hypoglycaemia

*Note:* All HbA1c values given have been converted to mmol/mol for ease of comparison.

Abbreviations: CGM, continuous glucose monitoring; DPV, Diabetes-Patienten-Verlaufsdokumentation (registry including data from 480 diabetes care centres in Germany and Austria, including >85% of youth with T1D in Germany); IMD, Index of Multiple Deprivation; isCGM, intermittently scanned CGM; MDI, multiple daily injections; NPDA, National Paediatric Diabetes Audit (annual report published by the Royal College of Paediatrics and Child Health in the United Kingdom. All paediatric diabetes units in England and Wales are asked to contribute data); rtCGM, real-time CGM; SES, socioeconomic status; T1D, type 1 diabetes; T1DX, Type 1 Diabetes Exchange Registry (registry containing data from 73 US paediatric and adult diabetes clinics).

^a^Calculated from Supporting Information 4: Table S1 to exclude those ≥26.

^b^Calculated from Table 1 excluding those ≥26.

**Table 2:** CGM use and outcomes stratified by ethnicity.

Study (setting, study period)	Ethnicity	Proportion of the total population	CGM use		Outcomes		
			Proportion of group using CGM	Secondary outcomes	Mean HbA1c of CGM users (mmol/mol)	Mean HbA1c of the whole cohort (mmol/mol)	Secondary outcomes
Addala (T1DX, 2010–2012) [19]	Non-Hispanic White	79.1%^a^	6.2%^a^	Minority status was not a significant predictor of CGM use on multivariable logistic regression modelling, adjusted for SES, sex, age and diabetes duration (*p*=0.972)	NA	66.6^a^	Minority status was a significant predictor of mean HbA1c on multiple logistic regression modelling, adjusted for SES, sex, age and diabetes duration (*p* < 0.001)
	Minority status	20.9%^a^	6.2%^a^		NA	69.2^a^	

Addala (T1DX, 2016–2018) [19]	Non-Hispanic White	77.7%^a^	38.4%^a^	Minority status was a significant predictor of CGM use on multivariable logistic regression modelling, adjusted for SES, sex, age and diabetes duration (*p* < 0.0001)	NA	68.5^a^	Minority status was a significant predictor of mean HbA1c on multiple logistic regression modelling, adjusted for SES, sex, age and diabetes duration (*p* < 0.001)
	Minority status	22.3%^a^	27.7%^a^		NA	73.3^a^	

Addala (DPV, 2010–2012) [19]	Non-Hispanic White	80.9%^a^	4.9%^a^	Minority status was a significant predictor of CGM use on multivariable logistic regression modelling, adjusted for SES, sex, age and diabetes duration (*p*=0.003)	NA	60.5^a^	Minority status was a significant predictor of mean HbA1c on multiple logistic regression modelling, adjusted for SES, sex, age and diabetes duration (*p* < 0.001)
	Minority status	19.1%^a^	3.7%^a^		NA	63^a^	

Addala (DPV, 2016–2018) [19]	Non-Hispanic White	76.1%^a^	56.6%^a^	Minority status was a significant predictor of CGM use on multivariable logistic regression modelling, adjusted for SES, sex, age and diabetes duration (*p* < 0.001)	NA	59.2^a^	Minority status was a significant predictor of mean HbA1c on multiple logistic regression modelling, adjusted for SES, sex, age and diabetes duration (*p* < 0.001)
	Minority status	23.9%^a^	47.5%^a^		NA	61.9^a^	

DeSalvo (T1DX, 2011) [21]	Non-Hispanic White	78.0%	4%	NA	NA	NA	NA
	Minority status	22.0%	2%		NA	NA	

DeSalvo (T1DX, 2016) [21]	Non-Hispanic White	78.0%	25%	NA	NA	NA	NA
	Minority status	22.0%	12%		NA	NA	

DeSalvo (DPV, 2011) [21]	Non-Hispanic White	80.0%	4%	NA	NA	NA	NA
	Minority status	20.0%	3%		NA	NA	

DeSalvo (DPV, 2016) [21]	Non-Hispanic White	77.0%	20%	NA	NA	NA	NA
	Minority status	23.0%	14%		NA	NA	

Foster (T1DX, 2016–2018) [22]	Non-Hispanic White	83.9%^b^	31.4^b^	NA	NA	NA	Mean HbA1c was significantly higher in Black than White or Hispanic participants across all age groups, even after adjusting for differences in SES and technology use on multivariate regression
	Non-Hispanic Black	5.7%^b^	7.6%^b^		NA	NA	
	Hispanic or Latino	10.4%^b^	20.7%^b^		NA	NA	

Wong (T1DX, 2010) [23]	Non-Hispanic White	80.3%^c^	5.3%^c^	For children <13 years of age, CGM was more frequent in non-Hispanic Whites than other races/ethnicities (chi-squared, *p* < 0.001), but this was not the case for children 13–18 (chi-squared, *p*=0.48) or young people 18–26 (chi-squared, *p*=0.18)	NA	NA	NA
	Non-Hispanic Black	5.1%^c^	2.3%^c^		NA	NA	
	Hispanic or Latino	9.5%^c^	3.5%^c^		NA	NA	
	Other	5.1%^c^	3.4%^c^		NA	NA	

Sawyer (US single centre, 2018–2020) [24]	Non-Hispanic White	64.3%	71%^d^	Hispanic and Black patients were significantly more likely to use no technology than be in the pump/CGM use group (ANOVA, *p* < 0.001)	NA	NA	NA
	Non-Hispanic Black	3.7%	42.3%^d^		NA	NA	
	Hispanic	15.5%	50.1%^d^		NA	NA	
	Other/unknown	16.4%	61.9%^d^		NA	NA	

Alonso (US single centre, 2016–2017) [25]	Non-Hispanic White	67.6%	45.4%	NA	NA	NA	NA
	Hispanic	17.2%	17.1%		NA	NA	
	Non-Hispanic Black	3.6%	21.2%		NA	NA	
	Other	11.6%	37.9%		NA	NA	

Alonso (US single centre, 2020–2021) [ 25]	Non-Hispanic White	67.6%	76.2%^e^	NA	NA	NA	NA
	Hispanic	17.2%	60.2%^e^		NA	NA	
	Non-Hispanic Black	3.6%	48.1%^e^		NA	NA	
	Other	11.6%	75.1%^e^		NA	NA	

Ravi (US single centre, 2015–2017) [26]	Non-Hispanic White	45.2%	27.5%	Participants with CGM exposure were more likely to be non-Hispanic White (chi-squared, *p* < 0.001) and less likely to be Hispanic (chi-squared, *p* < 0.001) than nonusers. There was no significant difference for Black (*p*=0.07), Asian (*p*=0.15) or other ethnic groups. On logistic regression predicting successful use of CGM, ethnicity was significantly associated with CGM use ≥85% (*p*=0.013). Hispanic participants were 66.5% less likely to successfully use CGM compared to non-Hispanic Whites	NA	NA	NA
	Hispanic	34.1%	13.2%		NA	NA	
	Black	7.2%	10.9%		NA	NA	
	American Indian	1.0%	0%		NA	NA	
	Asian	1.0%	42.9%		NA	NA	
	Mixed Race	2.5%	18.2%		NA	NA	
	Other/unknown	9.2%	14.6%		NA	NA	

Tremblay (US single centre, 2016–2020) [27]	White	70.7%	70.8%^f^	Non-White ethnicity was significantly associated with longer time to start CGM (Kaplan-Meier, *p*=0.0013) but not significantly associated with lower CGM uptake at 1 year (chi-squared, *p*=0.141). Hazard ratios for starting CGM within 1 year of diagnosis relative to White participants were 0.62 (0.45, 0.85) for Hispanic and 0.64 (0.44, 0.94) for Black participants.^g^ Non-White ethnicity was associated with higher discontinuation rates (chi-squared, *p* < 0.0001) and fewer days of use of CGM (Kruskal–Wallis, *p* < 0.0001)	NA	NA	NA
	Black	5.5%	62.2%^f^		NA	NA	
	Hispanic	8.7%	57.8%^f^		NA	NA	
	Other	7.2%	66.1%^f^		NA	NA	
	Unknown	7.9%	73.4%^f^		NA	NA	

Lee (US single centre, 2019) [28]	White	85.0%	62%	NA	67.2	72.7	There was a significant difference in mean HbA1c based on ethnicity (univariable regression, *p* < 0.001). In subgroup analysis of patients not using CGM, White participants had significantly lower HbA1c than Black participants (univariable regression *p*=0.002: mean HbA1c White 81.4; Black 93.4). Likewise, Black ethnicity was a significant predictor of higher HbA1c on multivariable regression, adjusted for age, insurance, sex and education (*p* < 0.001, intercept 1.2% [0.48, 1.77]). However, for patients using CGM, HbA1c did not significantly differ between ethnic groups (univariable regression, *p*=0.33). Likewise, Black ethnicity was not significantly predictive of higher HbA1c on multivariable regression, adjusted as above (*p*=0.21, intercept 0.42% [−0.24, 1.09]). There was no significant difference in TIR between ethnic groups (univariable regression, *p*=0.86; mean TIR White 40.4%; Black 43.0%; other 40.4%). Multivariable regression, adjusted as above, similarly showed no association between TIR and ethnicity (*p*=0.54, intercept 2.83% [−6.31, 1.20])
	Black	5.4%	30.3%		72.7	88	
	Other	9.6%	50.9%		67.2	77	

Lipman (US single centre, 2018–2019) [29]	Non-Hispanic White	77.0%	68%	White ethnicity was significantly associated with greater CGM use relative to Black (chi-squared, *p* < 0.001) and Hispanic (chi-squared, *p*=0.002) children. Adjusted odds ratio for not using CGM (adjusted for age and diabetes duration) were 3.4 (2.5, 4.7) for non-Hispanic Black and 1.9 (1.3, 2.9) for Hispanic children. Subgroup analysis demonstrated ethnic inequalities in CGM use for both those with government insurance (Black OR = 2.5 [1.6, 4.1], Hispanic OR = 1.5 [0.8, 2.7]) and commercial insurance (Black OR = 3.0 [1.9, 4.9], Hispanic OR = 1.6 [0.9, 2.9])	NA	61.7 (median)	NHW ethnicity was associated with lower median HbA1c relative to both Hispanic and NHB ethnicity (ANOVA, *p* < 0.001). Subgroup analysis showed the same association irrespective of insurance status. NHB children had the highest adjusted odds of having HbA1c measurement ≥58.5 mmol/mol (OR 4.9 [3.1–7.7] compared to NHW). This disparity was greater amongst the commercially insured (OR 5.1 [2.6–10.1]) than privately insured (OR 2.7 [1.4–5.4]). Hispanic children also had greater adjusted odds of failing to meet HbA1c targets (OR 2.7 [1.6, 4.4]). This was seen in both those with commercial (OR 1.8 [1.0–4.5]) and government insurance (OR 2.5 [1.0, 6.5])
	Non-Hispanic Black	15.0%	39%		NA	79.2 (median)	
	Hispanic	8.0%	53%		NA	70.5 (median)	

Lai (US single centre, 2015–2018) [30]	Non-Hispanic White	73.2%	54.3%	NHW ethnicity was associated with higher initiation rates of CGM compared to NHB (chi-squared, *p* < 0.001). On multivariate binomial logistic regression (adjusted for insurance type, age at diagnosis and sex), this association remained significant; NHB: B = −0.8, SE 0.2, OR = 0.5 (0.3, 0.6), *p* < 0.001; Hispanic: B = −0.7, SE 0.2, OR = 0.5 (0.3, 0.8), *p* < 0.001. NHW ethnicity was associated with higher continued use of CGM at 1 year (86%) relative to NHB (61%; OR 4.1 [2.1, 7.7]) but not Hispanic children (85%; OR 1.1 [0.3, 3.8]). On multivariate binomial logistic regression (adjusted as above), this association remained significant for NHB children (B = −1.4, SE 0.3, OR = 0.3 [0.1, 0.4], *p* < 0.001) and not significant for Hispanic children (B = −0.1, SE 0.5, OR = 0.9 [0.3, 2.5], *p*=0.8)	NA	NA	NA
	Non-Hispanic Black	18.5%	30.5%		NA	NA	
	Hispanic	8.3%	32.8%		NA	NA	

Choudhary (US single centre, 2019–2021) [31]	White	53.3%	NA	NA	NA	NA	On generalised linear modelling, adjusted for age, sex, insurance status and CGM use, Black and Hispanic ethnicity were significant predictors of higher HbA1c relative to White ethnicity (Black: estimated intercept HbA1c 1.31 [SE 0.09, *p* < 0.0001]; Hispanic; estimated intercept HbA1c 0.41 [SE 0.08, *p* < 0.0001]). Other ethnicity was not significantly associated with HbA1c. On generalised linear modelling, adjusted as above, Black and Hispanic ethnicity were significant predictors of lower TIR relative to White ethnicity (Black: estimated intercept TIR −7.60 [SE 1.49, *p* < 0.0001]; Hispanic: estimated intercept TIR −2.96 [SE 1.40, *p*=0.03]. Other ethnicity was not significantly associated with TIR
	Hispanic	22.3%	NA		NA	NA	
	Black	17.7%	NA		NA	NA	
	Other	6.7%	NA		NA	NA	

Sheikh (US single centre, 2015–2016) [32]	Non-Hispanic White	52.4%	27%	NHW ethnicity was significantly associated with higher rates of CGM use (Fisher's exact test, *p* < 0.001). On multiple logistic regression modelling, adjusted for sex, insurance status and primary language, this relationship maintained significance. Adjusted odds ratios of not using CGM were 1.62 (1.09, 2.40) for Hispanic and 4.06 (2.48, 6.66) for NHB patients	NA	69.4^h^	In a general linear model (least square means, adjusted for sex, insurance, language, pump and CGM use), NHW ethnicity was a significant predictor of lower HbA1c (*p* < 0.001): NHW HbA1c estimate 8.5% (SE 0.19), NHB HbA1c estimate 9.6% (SE 0.20), Hispanic HbA1c estimate 8.9% (SE 0.18), other HbA1c estimate 8.6% (SE 0.23)
	Non-Hispanic Black	16.8%	6%		NA	81.4^h^	
	Hispanic	24.3%	8.3%		NA	73.8^h^	
	Other	6.6%	20.6%		NA	70.5^h^	

Burnside (New Zealand, 2021) [2]	European/other	70.3%	74.9%	Lower rates of CGM use were significantly associated with Māori and Pacific ethnicity. Unadjusted risk ratios (univariate regression) for CGM use: Māori RR 1; Pacific RR 0.47 (0.30, 0.74); Asian RR 1.19 (0.91, 1.56); European/other RR 1.36 (1.17, 1.58). This association was maintained when adjusted for age, gender, deprivation quintile, diabetes duration, pump use and district health board on multivariable generalised regression analysis. Adjusted risk ratios for CGM use: Māori RR 1; Pacific RR 0.62 (0.40, 0.96); Asian RR 1.24 (0.96, 1.60); European/other RR 1.20 (1.04, 1.38). Ethnicity was a significant predictor for CGM use in both unadjusted and adjusted models (Wald test, *p* < 0.001)	64.1 (isCGM) 59.3 (rtCGM)	NA	Ethnicity was a significant predictor of HbA1c on both univariable and multivariable (adjusted for age, gender, deprivation quintile, diabetes duration, CGM use, pump use and district health board) linear regression models. Subgroup analysis demonstrated ethnicity was a significant predictor of HbA1c on adjusted and unadjusted models for children using SMBG or isCGM. However, ethnicity was not a significant predictor of outcomes for children using rtCGM. The difference in outcomes for those using rtCGM vs. SMBG was greatest for those of Māori ethnicity (adjusted difference −15.3 [−21.5, −9.1]). Mean HbA1c of children not using CGM: Māori 83.5 (SD 19.3); Pacific 78.4 (SD 18.8); Asian 63.2 (SD 13.0); European/other 68.7 (SD 16.3). Mean HbA1c of children using isCGM: Māori 71.9 (SD 16.8); Pacific 75.5 (SD 15.4); Asian 65.6 (SD 14.8); European/other 64.1 (SD 13.2). Mean HbA1c of children using rtCGM: Māori 61.8 (SD 10.9); Pacific 69.9 (SD 14.2); Asian 55.2 (SD 9.4); European/other 59.3 (SD 10.5)
	Māori	18.1%	55%		71.9 (isCGM) 61.8 (rtCGM)	NA	
	Pacific	7.1%	25.9%		75.5 (isCGM) 69.9 (rtCGM)	NA	
	Asian	4.6%	65.5%		65.6 (isCGM) 55.2 (rtCGM)	NA	

NPDA (England and Wales, 2021–2022) [12]	White	77.4%	30.8%	NA	NA	63.4	NA
	Asian	7.9%	25.4%		NA	65	
	Black	4.5%	22.2%		NA	70.7	
	Mixed	3.3%	31.3%		NA	66.4	
	Other	2.6%	26.9%		NA	62.7	

NPDA (England and Wales, 2020–2021) [41]	White	86.0%	28.4%	NA	NA	63.9	NA
	Asian	7.5%	25.3%		NA	65.2	
	Black	3.3%	21.9%		NA	70.9	
	Mixed	2.2%	29.3%		NA	67.2	
	Other	1.0%	28.1%		NA	63.3	

NPDA (England and Wales, 2019–2020) [42]	White	84.1%	20.2%	NA	NA	64.6	NA
	Asian	6.5%	15.1%		NA	65.8	
	Black	4.0%	11.7%		NA	71.9	
	Mixed	3.2%	18.5%		NA	67.4	
	Other	2.1%	16%		NA	63	

NPDA (England and Wales, 2018–2019) [43]	White	84.4%	13.5%	NA	NA	64.6	NA
	Asian	6.7%	6.7%		NA	66.2	
	Black	3.9%	6.5%		NA	71.4	
	Mixed	3.1%	11.6%		NA	67.5	
	Other	2.0%	10%		NA	63.7	

NPDA (England and Wales, 2017–2018) [44]	White	84.9%	9.9%	NA	NA	67	NA
	Asian	5.9%	4.9%		NA	68.8	
	Black	3.9%	4.1%		NA	74.9	
	Mixed	2.8%	7.9%		NA	69.6	
	Other	2.5%	6.1%		NA	67	

^a^Adjusted mean estimate from regression models including minority status, period, sex, age, diabetes duration, SES, minority status by period interaction and minority status by SES interaction.

^b^Calculated from Supporting Information 4: Table S3. The number of patients was estimated by summing (*n*/[%]) for each category, as over 6000 patients were missing data on household income.

^c^Calculated from Table 1 to exclude those ≥26.

^d^Calculated from Table 1 by combining those in MDI/CGM and pump/CGM categories.

^e^Calculated from Supporting Information 5: Table by combining those in MDI/CGM, pump/CGM and HCL categories.

^f^Started CGM within 1 year of diagnosis.

^g^These data refer to a group of meaningful CGM users followed up until 1 year after commencing meaningful use. The study period is slightly different to the rest of the data (January 2015 to March 2021).

^h^HbA1c values given here are estimates generated by a general linear model (least square means).

**Table 3:** CGM use and outcomes stratified by socioeconomic status and insurance status.

Measure of SES	Study (setting, study period)	SES/insurance group	Proportion of the total population	CGM use		Outcomes		
				Proportion of group using CGM	Secondary outcomes	Mean HbA1c of CGM users (mmol/mol)	Mean HbA1c of the whole cohort (mmol/mol)	Secondary outcomes
Household income	Addala (T1DX, 2010–2012) [19]	Annual household income >$100,000	NA	9.1%^a^	CGM use was estimated from a logistic regression model, adjusted for minority status, sex, age and diabetes duration. There was a significant association between CGM use and household income (*p* < 0.001). Slope index of inequality 0.381 (*p* < 0.001)	NA	63.7^a^	Mean HbA1c was estimated from a multiple logistic regression model, adjusted for minority status, sex, age, diabetes duration and technology use. There was a significant association between mean HbA1c and household income (*p* < 0.001). Slope index of inequality −0.301 (*p* < 0.001). When adjusted for pump and CGM use, the slope decreased to −0.255 (*p* < 0.001)
		Annual household income $75−100,000	NA	6.8%^a^		NA	66.9^a^	
		Annual household income $50−75,000	NA	4.8%^a^		NA	68.6^a^	
		Annual household income $35−50,000	NA	4.0%^a^		NA	72.2^a^	
		Annual household income $25−35,000	NA	4.2%^a^		NA	74.4^a^	
		Annual household income <$25,000	NA	3.5%^a^		NA	75.7^a^	
	Addala (T1DX, 2016–2018) [19]	Annual household income > $100,000	NA	49.1%^a^	CGM use was estimated from a logistic regression model, adjusted for minority status, sex, age and diabetes duration. There was a significant association between CGM use and household income (*p* < 0.001). Slope index of inequality 0.460 (*p* < 0.001). This was a significant increase in SII from 2010 to 2012 (*p*=0.0382)	NA	63.2^a^	Mean HbA1c was estimated from a multiple logistic regression model, adjusted for minority status, sex, age, diabetes duration and technology use. There was a significant association between mean HbA1c and household income (*p* < 0.001). Slope index of inequality −0.354 (*p* < 0.001). When adjusted for pump and CGM use, the slope decreased to −0.276 (*p* < 0.001). This was a significant increase in SII compared to 2010–2012 (*p*=0.0005), though the change was no longer significant if pump and CGM use were adjusted for (*p*=0.168)
		Annual household income $75−100,000	NA	40.7%^a^		NA	65.4^a^	
		Annual household income $50−75,000	NA	32.2%^a^		NA	69.2^a^	
		Annual household income $35−50,000	NA	21.8%^a^		NA	75.4^a^	
		Annual household income $25−35,000	NA	17.3%^a^		NA	76.9^a^	
		Annual household income <$25,000	NA	12.3%^a^		NA	74.2^a^	
	Foster (T1DX, 2016–2018) [22]	Annual household income ≥$75,000	52.6%^b^	37.6%^b^	NA	NA	NA	NA
		Annual household income $50,000-$75,000	16.4%^b^	26.5%^b^		NA	NA	
		Annual household income <$50,000	31%^b^	15.5%^b^		NA	NA	
	Wong (T1DX, 2010) [23]	Annual household income ≥$75,000	52.4%^c^	6.1%^c^	CGM use was more likely in patients with higher household income for children <18 (chi-squared, *p* < 0.001) but not for 18–26-year-olds (*p*=0.32)	NA	NA	NA
		Annual household income $35,000-$75,000	27.4%^c^	4.1%^c^		NA	NA	
		Annual household income <$35,000	20.2%^c^	2.4%^c^		NA	NA	

Index of Multiple Deprivation	Addala (DPV, 2010–2012) [19]	Least deprived quintile (income deprivation)	NA	3.5%^a^	CGM use was estimated from a logistic regression model, adjusted for minority status, sex, age and diabetes duration. There was a significant association between CGM use and income deprivation (*p* < 0.001). Slope index of inequality −0.053 (*p*=0.04)	NA	59.5^a^	Mean HbA1c was estimated from a multiple logistic regression model, adjusted for minority status, sex, age, diabetes duration and technology use. There was a significant association between mean HbA1c and income deprivation (*p* < 0.001). Slope index of inequality −0.093 (*p* < 0.001). When adjusted for pump and CGM use, the slope increased to −0.094 (*p* < 0.001)
		Second least deprived quintile (income deprivation)	NA	3.2%^a^		NA	61.1^a^	
		Third least deprived quintile (income deprivation)	NA	5.3%^a^		NA	62.3^a^	
		Second most deprived quintile (income deprivation)	NA	4.4%^a^		NA	63.5^a^	
		Most deprived quintile (income deprivation)	NA	5.5%^a^		NA	64.2^a^	
	Addala (DPV, 2016–2018) [19]	Least deprived quintile (income deprivation)	NA	55.6%^a^	CGM use was estimated from a logistic regression model, adjusted for minority status, sex, age and diabetes duration. There was a significant association between CGM use and income deprivation (*p* < 0.001). Slope index of inequality 0.068 (*p* < 0.001). This was a significant increase in SII from 2010 to 2012 (*p* < 0.0001)	NA	58.7^a^	Mean HbA1c was estimated from a multiple logistic regression model, adjusted for minority status, sex, age, diabetes duration and technology use. There was a significant association between mean HbA1c and income deprivation (*p* < 0.001). Slope index of inequality −0.078 (*p* < 0.001). When adjusted for pump and CGM use, the slope decreased to −0.074 (*p* < 0.001). There was a nonsignificant decrease in SII compared to 2010–2012 (*p*=0.0835)
		Second least deprived quintile (income deprivation)	NA	50.8%^a^		NA	59^a^	
		Third least deprived quintile (income deprivation)	NA	49.5% ^a^		NA	61^a^	
		Second most deprived quintile (income deprivation)	NA	54.6% ^a^		NA	62.4^a^	
		Most deprived quintile (income deprivation)	NA	49.6% ^a^		NA	62.6^a^	
	Auzanneau, 2018 (DPV, 2015–2016) [33]	Least deprived quintile (German Index of Multiple Deprivation)	24.3%^k^	6.3%^a^	Adjusted mean estimates, derived from logistic multivariate regression (least square means, adjusted for sex, age, migration background, German federal state and diabetes duration), showed significant differences (*p*=0.002) in CGM usage between SES quintiles	NA	62.2^j^	Adjusted mean estimates, derived from linear multivariate regression (least square means, adjusted for sex, age, migration background, German federal state and diabetes duration), showed significant differences (*p* < 0.001) in HbA1c between SES quintiles. Adjusted mean estimates, derived from linear multivariate regression (least square means, adjusted as above), showed significantly lower rates of severe hypoglycaemia in the most deprived quintiles (*p*=0.003). Adjusted mean estimates, derived from linear multivariate regression (least square means, adjusted as above), showed no significant difference in rates of DKA with deprivation quintiles as the independent variable (*p*=0.34)
		Second least deprived quintile	25.8%^k^	5.6%^k^		NA	62.3^j^	
		Third least deprived quintile	18.3%^k^	5.7%^k^		NA	63^j^	
		Second most deprived quintile	19.8%^k^	5.3%^k^		NA	63.5^j^	
		Most deprived quintile	11.9%^k^	3.4%^k^		NA	64.7^j^	
	Auzanneau, 2021 (DPV, 2016) [34]	Least deprived quintile (German Index of Multiple Deprivation)	NA	19.6%^l^	There was a significant difference in CGM use between the least and most deprived quintile on multivariate logistic regression, adjusting for sex, migration background, age and diabetes duration. OR for not using CGM 1.85 [1.63, 2.10] (*p* < 0.0001)	NA	NA	NA
		Second least deprived quintile	NA	14.6%^l^		NA	NA	
		Third least deprived quintile	NA	15.7%^l^		NA	NA	
		Second most deprived quintile	NA	13.9% ^l^		NA	NA	
		Most deprived quintile	NA	11.8% ^l^		NA	NA	
	Auzanneau, 2021 (DPV, 2017) [34]	Least deprived quintile (German Index of Multiple Deprivation)	NA	44.6%^l^	There was a significant difference in CGM use between the least and most deprived quintile on multivariate logistic regression, adjusting for sex, migration background, age and diabetes duration. OR for not using CGM 1.65 (1.49, 1.82) (*p* < 0.0001)	NA	NA	NA
		Second least deprived quintile	NA	37.1%^l^		NA	NA	
		Third least deprived quintile	NA	37.1%^l^		NA	NA	
		Second most deprived quintile	NA	38.2%^l^		NA	NA	
		Most deprived quintile	NA	33.2% ^l^		NA	NA	
	Auzanneau, 2021 (DPV, 2018) [34]	Least deprived quintile (German Index of Multiple Deprivation)	NA	60.4%^l^	There was a significant difference in CGM use between the least and most deprived quintile on multivariate logistic regression, adjusting for sex, migration background, age and diabetes duration. OR for not using CGM 1.52 (1.37, 1.67) (*p* < 0.0001)	NA	NA	NA
		Second least deprived quintile	NA	56.1%^l^		NA	NA	
		Third least deprived quintile	NA	56.1%^l^		NA	NA	
		Second most deprived quintile	NA	55.4%^l^		NA	NA	
		Most deprived quintile	NA	50.4%^l^		NA	NA	
	Auzanneau, 2021 (DPV, 2019) [34]	Least deprived quintile (German Index of Multiple Deprivation)	NA	69.3%^l^	There was no significant difference in CGM use between the least and most deprived quintile on multivariate logistic regression, adjusting for sex, migration background, age and diabetes duration. OR for not using CGM 0.97 (0.88, 1.08) (*p*=0.460)	NA	NA	NA
		Second least deprived quintile	NA	67.5%^l^		NA	NA	
		Third least deprived quintile	NA	65.7%^l^		NA	NA	
		Second most deprived quintile	NA	67.1%^l^		NA	NA	
		Most deprived quintile	NA	70.4%^l^		NA	NA	
	Stanley (Canada single centre, 2018–2020) [36]	Least deprived quintile (material deprivation dimension of the Ontario Marginalisation Index)	23.1%	59.0%	Material deprivation quintile was significantly associated with any CGM use (chi-squared, *p* < 0.0001). Unadjusted odds ratio of using CGM (Q1 vs. Q5) 2.68. rtCGM use was significantly associated with material deprivation quintile (chi-squared, *p* < 0.0001). Unadjusted odds ratio of using rtCGM (Q1 vs. Q5) 1.89. However, isCGM use was not associated with material deprivation quintile (chi-squared, *p*=0.724); in fact, higher odds of isCGM use were seen in the most compared to the least deprived quintile (*p*=0.006)	NA	67.2	Material deprivation quintile was significantly associated with mean HbA1c (one-way ANOVA, *p* < 0.0001). This association remained significant when adjusting for age, gender and diabetes duration on multivariable linear regression (ΔQ5–Q1 = 1.17 [0.82–1.52], *p* < 0.0001). rtCGM use was a significant partial mediator of the relationship between material deprivation quintile and HbA1c. The indirect effect of rtCGM use on HbA1c was estimated using Bayesian Monte Carlo Estimation; rtCGM use explained a HbA1c difference of 0.14% (0.06, 0.24), *p* < 0.0001, representing 12.0% of the difference in HbA1c between the most and least deprived quintiles. The coefficients for CGM use (all types) and isCGM use were not significant when assessed alongside MD quintiles
		Second least deprived quintile	20.7%	51.2%		NA	67.7	
		Third least deprived quintile	16%	43.1%		NA	69.8	
		Second most deprived quintile	16.4%	43.6%		NA	72.3	
		Most deprived quintile	23.9%	35.1%		NA	79.8	
	Ladd (Canada single centre, 2009–2021) [37]	Least deprived two quintiles (economic dependency)	55.5%	81.2%	There is no significant difference in CGM use between economic dependency quintiles (chi square, *p*=0.96). Likewise, there was no significant association on multivariable logistic regression, adjusted for age, sex, baseline HbA1c, pump use and diagnosis era (adjusted OR for CGM use [least vs. most deprived two quintiles]: 1.07 [0.60, 1.91])	NA	NA	There was no significant difference between quintiles in the proportion of patients achieving an improvement in HbA1c in the first 12 months after starting CGM (chi-square, *p*=0.35). Improvement in HbA1c (pre-CGM HbA1c minus post-CGM HbA1c) was not significantly associated with economic dependency on multivariable linear regression
		Third least deprived (economic dependency)	16.8%	80.0%		NA	NA	
		Most deprived two quintiles (economic dependency)	27.7%	80.6%		NA	NA	
	Burnside (New Zealand, 2021) [2]	Least deprived quintile (New Zealand Index of Deprivation)	23.2%	82.4%	Deprivation quintile was a significant predictor of CGM use on univariable generalised regression (Wald test, *p* < 0.001). Unadjusted risk ratios for CGM use: least deprived RR 1; second least RR 0.91 (0.81, 1.02); third least RR 0.87 (0.77, 0.98); second most RR 0.70 (0.60, 0.82); most deprived RR 0.54 (0.44, 0.66). This association was maintained, though less marked, on multivariable generalised regression, adjusted for age, ethnicity, deprivation quintile, diabetes duration, pump use and district health board (Wald test, *p* < 0.001). Adjusted risk ratios for CGM use: least deprived RR 1; second least RR 0.92 (0.83, 1.023); third least RR 0.91 (0.81, 1.02); second most RR 0.79 (0.68, 0.93); most deprived RR 0.69 (0.57, 0.84).	NA	62	Deprivation quintile was a significant predictor of HbA1c on univariable linear regression (Wald test, *p* < 0.001). Unadjusted HbA1c difference: least deprived 0; second least 2.5 (−0.9, 5.9); third least 2.4 (−1.1, 6.0); second most 7.3 (3.8, 10.9); most deprived 13.0 (9.4, 16.7). This association remained significant, though less marked, on multivariable linear regression adjusted for age, ethnicity, gender, diabetes duration, district health board, pump and CGM use (Wald test, *p*=0.012). Adjusted HbA1c difference: least deprived 0; second least 1.4 (−1.7, 4.5); third least 0.6 (−2.6, 3.9); second most 2.8 (−0.7, 6.2); most deprived 5.2 (1.5, 9.0)
		Second least deprived quintile	21.3%	75.0%		NA	64.5	
		Third least deprived quintile	19.1%	71.7%		NA	64.4	
		Second most deprived quintile	18.6%	58.0%		NA	69.3	
		Most deprived quintile	17.8%	44.4%		NA	75	
	Delagrange (France, 2017–2018) [40]	Least deprived quintile (European Deprivation Index)	19.5%	67.2%^m^	NA	NA	NA	Area deprivation was significantly associated with the proportion of patients with an HbA1c ≥8.5%. Percentage of patients with HbA1c ≥8.5%: least deprived 13.6%; second least 18.2%; third least 18.7%; second most 16.8%; most deprived 32.7%. On multivariable linear regression, adjusted for age, diabetes duration, gender, BMI and pump use, both area deprivation and individual deprivation were significant predictors of mean HbA1c. Individual deprivation: EPICES <30 ref; EPICES ≥30 *β* 0.43 (0.31, 0.55), *p* < 0.001 Area deprivation: least deprived ref; second least *β* 0.15 (−0.04, 0.34), *p*=0.124; third least *β* 0.14 (−0.04, 0.32), *p*=0.132; second most *β* 0.09 (−0.09, 0.28), *p*=0.326; most deprived *β* 0.38 (0.20, 0.55), *p* < 0.001. Higher individual level deprivation (EPICES score) was significantly associated with higher HbA1c on linear regression (*r* = 0.21)
		Second least deprived quintile	16.9%	67.0%^m^		NA	NA	
		Third least deprived quintile	20.3%	71.6%^m^		NA	NA	
		Second most deprived quintile	20.1%	73.1%^m^		NA	NA	
		Most deprived quintile	23.2%	69.8%^m^		NA	NA	
	NPDA (England and Wales, 2021–2022) [12]	Least deprived quintile (English and Welsh Index of Multiple Deprivation)	18.2%	37.7%	NA	NA	60.1	NA
		Second least deprived quintile	18.6%	32.7%		NA	61.8	
		Third least deprived quintile	19%	31.1%		NA	63.3	
		Second most deprived quintile	20.4%	25.7%		NA	65.1	
		Most deprived quintile	23.7%	23.9%		NA	67.6	
	NPDA (England and Wales, 2020–2021) [41]	Least deprived quintile (English and Welsh Index of Multiple Deprivation)	18.5%	34.7%	NA	NA	60.7	NA
		Second least deprived quintile	18.1%	29.8%		NA	62.3	
		Third least deprived quintile	19%	27.8%		NA	64.4	
		Second most deprived quintile	20.7%	24.9%		NA	65.2	
		Most deprived quintile	23.7%	23.8%		NA	67.8	
	NPDA (England and Wales, 2019–2020) [42]	Least deprived quintile (English and Welsh Index of Multiple Deprivation)	19%	25.2%	NA	NA	62	NA
		Second least deprived quintile	18.6%	22.3%		NA	62.7	
		Third least deprived quintile	19.1%	19.3%		NA	65.1	
		Second most deprived quintile	20.3%	17.3%		NA	66.1	
		Most deprived quintile	23%	14.0%		NA	68.3	
	NPDA (England and Wales, 2018–2019) [43]	Least deprived quintile (English and Welsh Index of Multiple Deprivation)	19.2%	16.1%	NA	NA	61.9	NA
		Second least deprived quintile	19.2%	16.5%		NA	63.4	
		Third least deprived quintile	18.9%	12.7%		NA	65	
		Second most deprived quintile	19.9%	10.3%		NA	66.2	
		Most deprived quintile	22.6%	8.2%		NA	68.2	
	NPDA (England and Wales, 2017–2018) [44]	Least deprived quintile (English and Welsh Index of Multiple Deprivation)	19.6%	12.8%	NA	NA	64.2	NA
		Second least deprived quintile	19.2%	11.3%		NA	65.9	
		Third least deprived quintile	18.9%	9.6%		NA	67.5	
		Second most deprived quintile	20%	7.3%		NA	68.7	
		Most deprived quintile	22.3%	6.1%		NA	70.9	

Insurance status	Addala (T1DX, 2010–2012) [19] — —	Private insurance	NA	6.8%^a^	CGM use was estimated from a logistic regression model adjusted for minority status, sex, age, diabetes duration, income, education and technology use. There was a significant association between CGM use and insurance status (*p* < 0.001)	NA	66.6^a^	CGM use was estimated from a logistic regression model adjusted for minority status, sex, age, diabetes duration, income, education and technology use. There was a significant association between mean HbA1c and insurance status (*p* < 0.001)
		Public insurance	NA	3.8%^a^		NA	73.9^a^	
		No insurance	NA	1.8%^a^		NA	69.6^a^	
	Addala (T1DX, 2016–2018) [19] — —	Private insurance	NA	39.8%^a^	CGM use was estimated from a logistic regression model adjusted for minority status, sex, age, diabetes duration, income, education and technology use. There was a significant association between CGM use and insurance status (*p* < 0.001)	NA	69^a^	CGM use was estimated from a logistic regression model adjusted for minority status, sex, age, diabetes duration, income, education and technology use. There was a significant association between mean HbA1c and insurance status (*p* < 0.001)
		Public insurance	NA	27.7%^a^		NA	77.5^a^	
		No insurance	NA	21.5%^a^		NA	72.9	
	Wong (T1DX, 2010) [23]	Private insurance	75.7%^c^	6.3%^c^	CGM use was more likely in patients with private insurance in all age groups (chi-squared, *p* < 0.001)	NA	NA	NA
		Other insurance	23.5%^c^	2.3%^c^		NA	NA	
		No insurance	0.1%^c^	2.2%^c^		NA	NA	
	Sawyer (US single centre, 2018–2020) [24]	Private insurance	60.3%	73.9%^d^	Patients with both private and Medicaid insurance were more likely to be in the pump/CGM group than in the no technology group (ANOVA, *p* < 0.001)	NA	NA	NA
		Medicaid	33.8%	50.3%^d^		NA	NA	
		Military plans	4.9%	68.9%^d^		NA	NA	
		Unknown	1.0%	NA		NA	NA	
	Alonso (US single centre, 2016–2017) [25]	Private insurance	63.6%	45.7%^e^	NA	NA	NA	NA
		Medicaid	31.7%	25.1%^e^		NA	NA	
		Military plans	3.6%	40.4%^e^		NA	NA	
		Other/none	1.1%	31.3%^e^		NA	NA	
	Alonso (US single centre, 2020–2021) [25]	Private insurance	64.6%	78.9%^e^	NA	NA	NA	NA
		Medicaid	31.3%	60.3%^e^		NA	NA	
		Military plans	3.5%	66.7%^e^		NA	NA	
		Other/none	0.5%	25.0%^e^		NA	NA	
	Tremblay (US single centre, 2016–2020) [27]	Private insurance	76.1%	71.5%^f^	Public insurance was associated with significantly lower uptake rates (chi-squared, *p*=0.0116) and longer time to start CGM (Kaplan–Meier, *p*=0.0058). Hazard ratios for starting CGM within 1 year of diagnosis relative to privately insured participants were 0.73 (0.59, 0.92) for public insured patients. Public insurance was further associated with higher discontinuation rates (chi-squared, *p*=0.004) and fewer days of use of CGM (Wilcoxon rank sum, *p* < 0.0001)^g^			NA
		Public insurance	23.9%	61.5%^f^		NA	NA	
	Lee (US single centre, 2019) [28]	Private insurance	77.6%	68.8%	NA	67.2	70.5	There was a significant difference in mean HbA1c based on insurance status (unpaired *t*-test, *p* < 0.001). In subgroup analyses of patients not using CGM, patients with public insurance had significantly higher HbA1c (unpaired *t*-test, *p* < 0.001: mean HbA1c private insurance 80.3; public insurance 88.0). This relationship remained significant on multivariable regression, adjusted for age, sex, ethnicity and education (*p* < 0.001, intercept 0.68% [0.29, 1.07]). However, insurance status was not significantly associated with HbA1c for patients using CGM (unpaired *t*-test, *p*=0.26. Multivariable regression, adjusted as above, similarly showed no significant association between HbA1c and insurance status (*p*=0.15, intercept 0.24% [−0.09, 0.57]). There was no significant difference in TIR based on insurance status (unpaired *t*-test, *p*=0.62; mean TIR private insurance 40.6%, public insurance 39.4%). Multivariable regression, adjusted as above, similarly showed no association between TIR and insurance status (*p*=0.61, intercept −1.25% [−6.07, 3.57])
		Public insurance	22.4%	32.8%		69.4	81.4	
	Lipman (US single centre, 2018–2019) [29]	Commercial insurance	73.0%^h^	68.1%^h^	NA	NA	NA	NA
		Government insurance	27.0%^h^	48.6%^h^		NA	NA	
	Lai (US single centre, 2015–2018) [30]	Commercial insurance	67.3%^i^	54.7%^i^	Commercial insurance was associated with higher initiation rates of CGM compared to government insurance (chi-squared, *p* < 0.001). On multivariate binomial logistic regression (adjusted for ethnicity, age at diagnosis and sex), this association remained significant; B = −0.6, SE 0.1, OR = 0.5 [0.4, 0.7], *p* < 0.001). On multivariate binomial logistic regression (adjusted as above), commercial insurance was not significantly associated with higher continued use of CGM at 1 year (B = −0.2, SE 0.3, OR = 0.8 [0.5, 1.3], *p*=0.4)	NA	NA	NA
		Government insurance	32.7% ^i^	34.5% ^i^		NA	NA	
	Choudhary (US single centre, 2019–2020) [31]	Commercial insurance	60.3%	61.8%	NA	NA	NA	Significantly more CYP with noncommercial insurance were hospitalised with DKA or severe hyperglycaemia than CYP with commercial insurance (16.1% vs. 5.8%, *p* < 0.0001)
		Noncommercial insurance	39.7%	24.5%		NA	NA	
	Choudhary (US single centre, 2020–2021) [31]	Commercial insurance	60.3%	61.4%	NA	NA	NA	On generalised linear modelling, adjusted for age, gender and ethnicity, noncommercial insurance was significantly associated with higher HbA1c (estimated intercept 0.62 (SE 0.07, *p* < 0.0001). On generalised linear modelling, adjusted as above, noncommercial insurance was significantly associated with lower TIR (estimated intercept −7.50 [SE 1.15, *p* < 0.0001]). Significantly more CYP with noncommercial insurance were hospitalised with DKA or severe hyperglycaemia than CYP with commercial insurance (16.1% vs. 6.9%, *p* < 0.0001)
		Noncommercial insurance	39.7%	35.7%		NA	NA	
	Sheikh (US single centre, 2015–2016) [32]	Private insurance	66.5%	26.6%	Private insurance was significantly associated with higher rates of CGM use (Fisher's exact test, *p* < 0.0001). On multiple logistic regression modelling, adjusted for sex, primary language and ethnicity, this relationship maintained significance. The adjusted odds ratio of not using CGM for patients with public insurance was 11.59 (6.72, 19.99)	NA	73.8^i^	In a general linear model (least square means, adjusted for ethnicity, sex, language, pump and CGM use), private insurance was significantly associated with lower HbA1c (*p*=0.003): private insurance HbA1c estimate 8.9% (SE 0.15); public insurance HbA1c estimate 9.2% (SE 0.16); no insurance HbA1c estimate 8.6% (SE 0.35)
		Public insurance	32.1%	2.3%		NA	77.0^i^	
		No insurance	1.4%	7.1%		NA	70.5^i^	

^a^Adjusted mean estimate from logistic (percentage using CGM) or linear (HbA1c) regression models including income or education or insurance and period, sex, age, diabetes duration, minority status, income or education or insurance by period interaction and income or education or insurance by minority status interaction.

^b^Calculated from Supporting Information 4: Table S3. The number of patients was estimated by summing (*n*/[%]) for each category, as over 6000 patients were missing data on household income.

^c^Calculated from Table 1 to exclude those ≥26.

^d^Calculated from Table 1 by combining those in MDI/CGM and pump/CGM categories.

^e^Calculated from Supporting Information 5: Table by combining those in MDI/CGM, pump/CGM and HCL categories.

^f^Started CGM within 1 year of diagnosis.

^g^These data refer to a group of meaningful CGM users followed up until 1 year after commencing meaningful use. The study period is slightly different to the rest of the data (January 2015 to March 2021).

^h^Calculated by summing ethnic group data in Table 2.

^i^Calculated by summing ethnic group data in Supporting Information 4: Table S1.

^j^HbA1c values given here are estimates generated by a general linear model (least square means).

^k^All values taken from model adjusted for sex, age group, migration, diabetes duration and federal state. HbA1c values for quintiles 2–4 not explicitly reported: read using inspect tool from Figure 2. Relevant data were calculated through combination of male and female data.

^l^Percentage using CGM not explicitly reported. All data included are estimates from logistic regression models, adjusted for area deprivation, migration background, gender, age group, diabetes duration and migration background—area deprivation interaction, and were read using inspect tool from Figure 2.

^m^Results read from Figure 4 using the inspect tool.

**Table 4:** CGM use and outcomes stratified by sex.

Study (setting, study period)	Sex	Proportion of the total population	CGM use	Outcomes			
			Proportion of group using CGM	Secondary outcomes	Mean HbA1c of CGM users (mmol/mol)	Mean HbA1c of the whole cohort (mmol/mol)	Secondary outcomes
DeSalvo (T1DX, 2011) [21]	Male	51%	3%	NA	NA	NA	NA
	Female	49%	3%		NA	NA	

DeSalvo (T1DX, 2016) [21]	Male	52%	21%	NA	NA	NA	NA
	Female	48%	22%		NA	NA	

DeSalvo (DPV, 2011) [21]	Male	52%	4%	NA	NA	NA	NA
	Female	48%	4%		NA	NA	

DeSalvo (DPV, 2016) [21]	Male	52%	18%	NA	NA	NA	NA
	Female	48%	19%		NA	NA	

Wong (T1DX, 2010) [23]	Male	51.2%^a^	4.4%^a^	There was no significant difference in CGM between males and females (chi-squared: *p*=0.25 for <13-year-olds; *p*=0.48 for 13–18-year-olds; *p*=0.03 for 18–26-year-olds)	NA	NA	NA
	Female	48.8%^a^	5.3%^a^		NA	NA	

Sawyer (US single centre, 2018–2020) [24]	Male	52.9%	63.6%^b^	NA	NA	NA	NA
	Female	47.1%	66.9%^b^		NA	NA	

Alonso (US single centre, 2016–2017) [25]	Male	52.3%	41.9% ^c^	NA	NA	NA	NA
	Female	47.7%	35.4%^c^		NA	NA	

Alonso (US single centre, 2020–2021) [25]	Male	52.3%	72.7%^c^	NA	NA	NA	NA
	Female	47.7%	71.9%^c^		NA	NA	

Ravi (US single centre, 2015–2017) [26]	Male	48.3%	19%	Sex was not significantly associated with CGM use (chi-squared, *p*=0.61)	NA	NA	NA
	Female	51.7%	20.6%		NA	NA	

Tremblay (US single centre, 2016–2020) [27]	Male	56.9%	67.7%^d^	There was no significant difference in CGM uptake (chi-squared, *p*=0.356), discontinuation rates (chi-squared, *p*=0.705) or days of use (Wilcoxon signed rank, *p*=0.931)^e^	NA	NA	NA
	Female	43.1%	70.9%^d^		NA	NA	

Lee (US single centre, 2019) [28]	Male	50.2%	58.7%	NA	66.1	73.8	There was no significant difference in mean HbA1c based on sex (unpaired *t*-test, *p*=0.53). In subgroup analysis of patients not using CGM, mean HbA1c did not significantly differ between sexes (unpaired *t*-test, *p*=0.09: mean HbA1c male 85.8; female 81.4). Likewise, sex was not a significant predictor of HbA1c on multivariable regression, adjusted for age, education, ethnicity and insurance, (*p*=0.11, intercept 0.31% [−0.07, 0.68]). For patients using CGM, HbA1c similarly did not significantly differ between sexes (unpaired *t*-test, *p*=0.27). Likewise, sex was not a significant predictor of HbA1c on multivariable regression, adjusted as above (*p*=0.50, intercept 0.07% [−0.14, 0.29]). There was no significant difference in TIR between sexes (unpaired *t*-test, *p*=0.32; mean TIR male 39.7%, female 41.3%). Multivariable regression, adjusted as above, similarly showed no association between TIR and sex (*p*=0.46, intercept 1.19% [−1.93, 4.30])
	Female	49.8%	59.7%		68.3	72.7	

Lai (US single centre, 2015–2018) [30]	Male	56.3%^f^	47.2%^f^	Logistic regression (adjusted for insurance, ethnicity, age at diagnosis) predicting likelihood of CGM initiation showed no significant association with sex: B = −0.1, SE 0.1, OR = 0.9 (0.7, 1.1) *p*=0.20. Logistic regression (adjusted as above) predicting likelihood of continued CGM use at 1 year showed no significant association with sex: B = −0.3, SE 0.2, OR = 0.8 (0.5, 1.2), *p*=0.2	NA	NA	NA
	Female	43.7%^f^	49.2%^f^		NA	NA	

Choudhary (US single centre, 2019–2021) [31]	Male	52.8%	NA	NA	NA	NA	On generalised linear modelling, adjusted for age, insurance status and ethnicity, female gender was not significantly associated with HbA1c (estimated intercept 0.11 (SE 0.06, *p*=0.06). On generalised linear modelling, adjusted as above, gender was not significantly associated with TIR
	Female	47.2%	NA		NA	NA	

Sheikh (US single centre, 2015–2016) [32]	Male	49.3%	16.8%	Sex was not significantly associated with CGM use (Fisher's exact test, *p*=0.057). However, on multiple logistic regression modelling, adjusted for ethnicity, insurance status and primary language, male sex was associated with significantly higher odds of not using CGM (OR: 1.32 [1.03, 1.69])	NA	73.8^g^	In a general linear model (least square means adjusted for ethnicity, insurance, language, pump and CGM use), there was no significant association between sex and HbA1c: female HbA1c estimate 8.9% (SE 0.18), male HbA1c estimate 8.9% (SE 0.19)
	Female	50.7%	20.2%		NA	73.8^g^	

Auzanneau, 2021 (DPV, 2016) [34]	Male	52.6%	15%^h^	There was no significant sex difference in CGM use on multivariate logistic regression, adjusting for area deprivation, migration background, age and diabetes duration. OR 1.05 (0.98, 1.12) (*p*=0.135)	NA	NA	NA
	Female	47.4%	15.7%^h^		NA	NA	

Auzanneau, 2021 (DPV, 2017) [34]	Male	52.6%	38.2%^h^	There was no significant sex difference in CGM use on multivariate logistic regression, adjusting for area deprivation, migration background, age and diabetes duration. OR 1.01 (0.96, 1.06) (*p*=0.746)	NA	NA	NA
	Female	47.4%	38.6%^h^		NA	NA	

Auzanneau, 2021 (DPV, 2018) [34]	Male	52.9%	55.7%^h^	There was no significant sex difference in CGM use on multivariate logistic regression, adjusting for area deprivation, migration background, age and diabetes duration. OR 1.02 (0.97, 1.08) (*p*=0.383)	NA	NA	NA
	Female	47.1%	56.4%^h^		NA	NA	

Auzanneau, 2021 (DPV, 2019) [34]	Male	52.8%	67.1%^h^	There was no significant sex difference in CGM use on multivariate logistic regression, adjusting for area deprivation, migration background, age and diabetes duration. OR 1.02 (0.97, 1.08) (*p*=0.451)	NA	NA	NA
	Female	47.2%	67.5%^h^		NA	NA	

Kordonouri (Germany single centre, 2017–18) [35]	Male	53.3%	32%	There was no significant sex difference in CGM use (chi-squared, *p* value not provided)	60	61	On multiple regression analysis, adjusted for age, diabetes duration and technology use, sex was not a significant predictor of HbA1c (beta = 0.01, *t* = 0.266, *p*=0.790)
	Female	46.7%	31.5%		60	61	

Ladd (Canada single centre, 2009–2021) [37]	Male	52.3%	76.8%	Female sex was significantly associated with higher rates of CGM use (chi-squared, *p*=0.01)	NA	NA	Improvement in HbA1c (pre-CGM HbA1c minus post-CGM HbA1c) was not significantly associated with sex on multivariable linear regression
	Female	47.3%	85.2%		NA	NA	

Bratke (Norwegian Childhood Diabetes Registry, 2017) [38]	Male	54.6%	49.3%^i^	NA	NA	NA	NA
	Female	45.4%	51.6%		NA	NA	

Burnside (New Zealand, 2021) [2]	Male	49.9%	66.4%	Sex was not a significant predictor of CGM use on univariable (Wald test, *p*=0.99) or multivariable generalised regression (Wald test, *p*=0.50), adjusted for age, ethnicity, deprivation quintile, diabetes duration, pump use and district health board. Unadjusted risk ratio for CGM use: female RR 1; male RR 0.97 (0.90, 1.05). Adjusted risk ratio for CGM use: female RR 1; male RR 1.00 (0.93, 1.07)	NA	66.1	Sex was not a significant predictor of HbA1c on univariable (mean HbA1c difference: female 0; male −1.2 [−3.0, 0.6], *p*=0.188) or multivariable linear regression models (mean HbA1c difference: female 0; male −1.5 [−3.2, 0.1], *p*=0.074), adjusted for age, ethnicity, deprivation, diabetes duration, district health board, pump and CGM use
	Female	50.1%	68.5%		NA	67.3	

Šumník (Czechia, 2017) [39]	Male	53%	52% of 0–5 s, 48% of 5–10 s, 35% of 10–15 s, 28% of 15+s	Female gender was significantly associated with higher rates of CGM use (chi-squared, *p* < 0.001) for all age categories but 5–10-year-olds. The same pattern was seen for rtCGM. However, there were no significant sex differences in isCGM use	NA	NA	NA
	Female	47%	55% of 0–5 s, 48% of 5–10 s, 40% of 10–15 s, 33% of 15+s		NA	NA	

Šumník (Czechia, 2018) [39]	Male	53%	69% of 0–5 s, 65% of 5–10 s, 48% of 10–15 s, 36% of 15+s	Female gender was significantly associated with higher rates of CGM use (chi-squared, *p* < 0.001) for all age categories but 5–10-year-olds. The same pattern was seen for rtCGM. However, there were no significant sex differences in isCGM use	NA	NA	NA
	Female	47%	72% of 0–5 s, 65% of 5–10 s, 54% of 10–15 s, 41% of 15+s		NA	NA	

Šumník (Czechia, 2019) [39]	Male	53%	81% of 0–5 s, 86% of 5–10 s, 77% of 10–15 s, 61% of 15+s	Female gender was significantly associated with higher rates of CGM use (chi-squared, *p* < 0.001) for all age categories but 5–10-year-olds. The same pattern was seen for rtCGM. However, there were no significant sex differences in isCGM use	NA	NA	NA
	Female	47%	95% of 0–5 s, 88% of 5–10 s, 78% of 10–15 s, 67% of 15+s		NA	NA	

^a^Calculated from Table 1 to exclude those ≥26.

^b^Calculated from Table 1 by combining those in MDI/CGM and pump/CGM categories.

^c^Calculated from Supporting Information 5: Table by combining those in MDI/CGM, pump/CGM and HCL categories.

^d^Started CGM within 1 year of diagnosis.

^e^These data refer to a group of meaningful CGM users followed up until 1 year after commencing meaningful use. The study period is slightly different to the rest of the data (January 2015 to March 2021).

^f^Calculated by summing ethnic group data in Supporting Information 4: Table S1.

^g^HbA1c values given here are estimates generated by a general linear model (least square means).

^h^Percentage using CGM not explicitly reported. All data included are estimates from logistic regression models, adjusted for area deprivation, migration background, gender, age group, diabetes duration and migration background—area deprivation interaction, and were read using inspect tool from Figure 2.

^i^Male data calculated from total patient numbers and female data in Table 1.

**Table 5:** CGM use and outcomes stratified by other PROGRESS-Plus criteria.

PROGRESS-Plus criterion assessed	Study (setting, study period)	Group	Proportion of the total population	CGM use		Outcomes		
				Proportion of group using CGM	Secondary outcomes	Mean HbA1c of CGM users (mmol/mol)	Mean HbA1c of the whole cohort (mmol/mol)	Secondary outcomes
Parental education	Addala (T1DX, 2010–2012) [19]	Professional or doctorate degree	NA	11.1^a^	CGM use was estimated from a logistic regression model adjusted for minority status, sex, age, diabetes duration, income, insurance and technology use. There was a significant association between CGM use and parental education (*p* < 0.001)	NA	62^a^	Mean HbA1c was estimated from a logistic regression model adjusted for minority status, sex, age, diabetes duration, income, insurance and technology use. There was a significant association between mean HbA1c and parental education (*p* < 0.001)
		Master's degree	NA	9.3^a^		NA	64.2^a^	
		Bachelor's degree	NA	6.4^a^		NA	66.6^a^	
		Associate's degree	NA	5.3^a^		NA	71.6^a^	
		High school graduate or GED	NA	2.9^a^		NA	73.2^a^	
		<High school diploma	NA	3.1^a^		NA	74.4^a^	
	Addala (T1DX, 2016–2018) [19]	Professional or doctorate degree	NA	53.9^a^	CGM use was estimated from a logistic regression model adjusted for minority status, sex, age, diabetes duration, income, insurance and technology use. There was a significant association between CGM use and parental education (*p* < 0.001)	NA	63.2^a^	Mean HbA1c was estimated from a logistic regression model adjusted for minority status, sex, age, diabetes duration, income, insurance and technology use. There was a significant association between mean HbA1c and parental education (*p* < 0.001)
		Master's degree	NA	43.0^a^		NA	65.4^a^	
		Bachelor's degree	NA	39.3^a^		NA	69.2^a^	
		Associate's degree	NA	27.7 ^a^		NA	75.4^a^	
		High school graduate or GED	NA	19.3 ^a^		NA	76.9^a^	
		<High school diploma	NA	21.6^a^		NA	73.1^a^	
	Addala (DPV, 2010–2012) [19]	Least deprived quintile (education deprivation)	NA	6.3^a^	CGM use was estimated from a logistic regression model adjusted for minority status, sex, age, diabetes duration, income deprivation and technology use. There was a significant association between CGM use and parental education deprivation (*p* < 0.001)	NA	62.2^a^	Mean HbA1c was estimated from a logistic regression model adjusted for minority status, sex, age, diabetes duration, income deprivation and technology use. There was a significant association between mean HbA1c and parental education (*p*=0.036)
		Second least deprived quintile (education deprivation)	NA	4.4^a^		NA	61.6^a^	
		Third least deprived quintile (education deprivation)	NA	4.5^a^		NA	61.3^a^	
		Second most deprived quintile (education deprivation)	NA	3.5^a^		NA	61.6^a^	
		Most deprived quintile (education deprivation)	NA	3.2^a^		NA	61.1^a^	
	Addala (DPV, 2016–2018) [19]	Least deprived quintile (education deprivation)	NA	52.2^a^	CGM use was estimated from a logistic regression model adjusted for minority status, sex, age, diabetes duration, income deprivation and technology use. There was a significant association between CGM use and parental education deprivation (*p* < 0.001)	NA	60.9^a^	Mean HbA1c was estimated from a logistic regression model adjusted for minority status, sex, age, diabetes duration, income deprivation and technology use. There was a significant association between mean HbA1c and parental education (*p* < 0.001)
		Second least deprived quintile (education deprivation)	NA	59.1^a^		NA	61.4^a^	
		Third least deprived quintile (education deprivation)	NA	53.9^a^		NA	59.6^a^	
		Second most deprived quintile (education deprivation)	NA	46.6^a^		NA	59.6^a^	
		Most deprived quintile (education deprivation)	NA	47.0^a^		NA	59.5^a^	
	Wong (T1DX, 2010) [23]	Master's, professional, doctorate	24.4% ^b.^	7.9^b^	CGM use was more likely in patients with higher parental education level for children <18 (chi-squared, *p* < 0.001)	NA	NA	NA
		Associate's or bachelor's	31.6%^b.^	5.1^b^		NA	NA	
		≤ High school/GED	44.0%^b^	2.5^b^		NA	NA	
	Lee (US single centre, 2019) [28]	≥ College degree	47.4%	73.0	NA	65.0	68.3	There was a significant difference in mean HbA1c based on parental education (univariable regression, *p* < 0.001). In subgroup analysis of patients not using CGM, patients with higher parental education had significantly lower HbA1c (univariable regression, *p*=0.02; mean HbA1c for ≥ college education 77.0, < college education 86.9). This relationship remained significant on multivariable regression, adjusted for age, sex, ethnicity and insurance (*p* < 0.001, intercept 0.77% [0.35, 1.20]). Parental education was also significantly associated with HbA1c for patients using CGM (univariable regression, *p* < 0.001). Multivariable regression, adjusted as above, similarly showed a significant association between higher HbA1c and lower parental education (*p* < 0.001, intercept 0.52% [0.27, 0.76]). There was a significant difference in TIR based on parental education (univariable regression, *p*=0.009; mean TIR ≥ college education 42.3%, < college education 36.8%)
		< College degree	36.6%	44.5		70.5	79.2	
		Unknown	16.1%	52.3		68.3	76	

Primary language	Sawyer (US single centre, 2018–2020) [24]	English	94.9%^c^	66.8^c^	Spanish-speaking patients were significantly more likely to use no technology than be in any technology-using group (ANOVA, *p* < 0.001)	NA	NA	NA
		Spanish	4.4%^c^	34.1^c^		NA	NA	
		Other	0.9%^c^	46.4^c^		NA	NA	
	Tremblay (US single centre, 2016–2020) [27]	English	94.0%	69.7^d^	There was no significant difference in CGM uptake based on primary language (chi-squared, *p*=0.227). However, not speaking English as a first language was significantly associated with discontinuation of CGM (chi-squared, *p*=0.004) and use of CGM on fewer days (Kruskal–Wallis, *p*=0.0001)^e^	NA	NA	NA
		Spanish	3.4%	57.1^d^		NA	NA	
		Other	2.6%	61.9^d^		NA	NA	
	Sheikh (US single centre, 2015–2016) [32]	English	92.2%	20.0	Primary language of parents being English was significantly associated with higher rates of CGM use (Fisher's exact test, *p* < 0.0001). On multiple logistic regression modelling, adjusted for sex, insurance status and ethnicity, this relationship maintained significance. The adjusted odds ratio of not using CGM for patients with Spanish-speaking parents was 6.59 (1.51, 28.73)	NA	74.9^f^	In a general linear model (least square means, adjusted for ethnicity, insurance, sex, pump and CGM use), having English-speaking parents was not significantly associated with lower HbA1c (*p*=0.234): English-speaking HbA1c estimate 9.0% (SE 0.17); Spanish-speaking HbA1c estimate 8.8% (SE 0.23)
		Spanish	7.8%	1.3		NA	72.7^f^	

Place of residence	Sawyer (US single centre, 2018–2020) [24]	Urban	83.8%^c^	65.3^c^	Technology use did not significantly differ based on rural versus nonrural location (ANOVA)	NA	NA	NA
		Rural	16.2%^c^	64.6^c^		NA	NA	

Migration background	Auzanneau, 2021 (DPV, 2016) [34]	No migration background	77.4%	19.6^g^	There was a significant difference in CGM use between those with and without migration background on multivariate logistic regression, adjusting for sex, IMD quintile, age and diabetes duration. OR for not using CGM 1.79 (1.64, 1.95) (*p* < 0.0001)	NA	NA	NA
		Second generation immigrant	18.2%	11.8^g^		NA	NA	
		First generation immigrant	4.0%	10.7^g^		NA	NA	
	Auzanneau, 2021 (DPV, 2017) [34]	No migration background	76.5%	45.7^g^	There was a significant difference in CGM use between those with and without migration background on multivariate logistic regression, adjusting for sex, IMD quintile, age and diabetes duration. OR for not using CGM 1.76 (1.65, 1.87) (*p* < 0.0001)	NA	NA	NA
		Second generation immigrant	18.7%	33.2^g^		NA	NA	
		First generation immigrant	4.5%	24.3^g^		NA	NA	
	Auzanneau, 2021 (DPV, 2018) [34]	No migration background	76.1%	64.3^g^	There was a significant difference in CGM use between those with and without migration background on multivariate logistic regression, adjusting for sex, IMD quintile, age and diabetes duration. OR for not using CGM 1.44 (1.36, 1.53) (*p* < 0.0001)	NA	NA	NA
		Second generation immigrant	18.7%	54.3^g^		NA	NA	
		First generation immigrant	4.8%	44.3^g^		NA	NA	
	Auzanneau, 2021 (DPV, 2019) [34]	No migration background	75.7%	77.0^g^	There was a significant difference in CGM use between those with and without migration background on multivariate logistic regression, adjusting for sex, IMD quintile, age and diabetes duration. OR for not using CGM 1.30 (1.22, 1.39) (*p* < 0.0001)	NA	NA	NA
		Second generation immigrant	19.0%	67.1^g^		NA	NA	
		First generation immigrant	5.1%	58.9^g^		NA	NA	

Other	Ladd (Canada single centre, 2009–2021) [37]	Least deprived two quintiles (residential instability)	50.3%	84.3	There is no significant difference in CGM use between residential instability quintiles (chi-squared, *p*=0.10). Likewise, there was no significant association on multivariable logistic regression, adjusted for age, sex, baseline HbA1c, pump use and diagnosis era (adjusted OR for CGM use (least vs. most deprived two quintiles): 0.95 [0.52, 1.73])	NA	NA	There was no significant difference between quintiles in the proportion of patients achieving an improvement in HbA1c in the first 12 months after starting CGM (chi-squared, *p*=0.72). Improvement in HbA1c (pre-CGM HbA1c minus post-CGM HbA1c) was not significantly associated with residential instability on multivariable linear regression
		Third least deprived (residential instability)	21.8%	77.7		NA	NA	
		Most deprived two quintiles (residential instability)	27.9%	77.1		NA	NA	
	Ladd (Canada single centre, 2009–2021) [37]	Least deprived two quintiles (ethnocultural composition)	34.8%	83.6	There is no significant difference in CGM use between ethnocultural composition quintiles (chi-squared, *p*=0.14). Likewise, there was no significant association on multivariable logistic regression, adjusted for age, sex, baseline HbA1c, pump use and diagnosis era (adjusted OR for CGM use (least vs. most deprived two quintiles): 0.73 [0.42, 1.27])	NA	NA	There was a significant difference between quintiles in the proportion of patients achieving an improvement in HbA1c in the first 12 months after starting CGM (chi-squared, *p*=0.002). More patients in the most deprived 2 quintiles achieved an improvement (53.6%) than in the least deprived 2 quintiles (35.0%). When analysis was limited to those with diabetes ≥1 year, this difference was no longer significant (chi-squared, *p*=0.16). Improvement in HbA1c (pre-CGM HbA1c minus post-CGM HbA1c) was not significantly associated with ethnocultural composition on multivariable linear regression
		Third least deprived (ethnocultural composition)	20.8%	83.9		NA	NA	
		Most deprived two quintiles (ethnocultural composition)	44.4%	77.3		NA	NA	
	Ladd (Canada single centre, 2009–2021) [22]	Least deprived two quintiles (situational vulnerability)	60.2%	83.5	There is no significant difference in CGM use between situational vulnerability quintiles (chi-squared, *p*=0.05). Likewise, there was no significant association on multivariable logistic regression, adjusted for age, sex, baseline HbA1c, pump use and diagnosis era (adjusted OR for CGM use (least vs. most deprived two quintiles): 0.56 [0.29, 1.08])	NA	NA	There was no significant difference between quintiles in the proportion of patients achieving an improvement in HbA1c in the first 12 months after starting CGM (chi-squared, *p*=0.13). Improvement in HbA1c (pre-CGM HbA1c minus post-CGM HbA1c) was not significantly associated with situational vulnerability on multivariable linear regression
		Third least deprived (situational vulnerability)	19.5%	80.2		NA	NA	
		Most deprived two quintiles (situational vulnerability)	20.3%	73.6		NA	NA	
	Tremblay (US single centre, 2016–2020) [33]	Two parents, one house	71.4%	75.3^d^	Family structure was significantly associated with starting CGM within a year of diagnosis (Kruskal–Wallis, *p*=0.002)	NA	NA	NA
		Two parents, two houses	12.8%	69.2^d^		NA	NA	
		One parent, one house	7.4%	60.0^d^		NA	NA	
		Non-parent primary care giver	2.0%	43.8^d^		NA	NA	
	Tremblay (US single centre, 2016–2020) [27]	Professional	44.3%	76.7^d^	Primary caregiver occupation was significantly associated with starting CGM within a year of diagnosis (Kruskal–Wallis, *p* < 0.0001)	NA	NA	NA
		Service/trade	10.3%	57.6^d^		NA	NA	
		Shift work	3.8%	79.2^d^		NA	NA	
		Stay at home parent	22.4%	79.0^d^		NA	NA	
		Technical/associated professional	14.9%	69.5^d^		NA	NA	
		Unemployed	4.4%	64.3^d^		NA	NA	

^a^Adjusted mean estimate from logistic (percentage using CGM) or linear (HbA1c) regression models including income or education or insurance and period, sex, age, diabetes duration, minority status, income or education or insurance by period interaction and income or education or insurance by minority status interaction.

^b^Calculated from Table 1 to exclude those ≥26.

^c^Calculated from Table 1 by combining those in MDI/CGM and pump/CGM categories.

^d^Started CGM within 1 year of diagnosis.

^e^These data refer to a group of meaningful CGM users followed up until 1 year after commencing meaningful use. The study period is slightly different to the rest of the data (January 2015 to March 2021).

^f^HbA1c values given here are estimates generated by a general linear model (least square means).

^g^Percentage using CGM not explicitly reported. All data included are estimates from logistic regression models, adjusted for area deprivation, migration background, gender, age group, diabetes duration and migration background—area deprivation interaction, and were read using inspect tool from Figure 2.

## Data Availability

The data that support the findings of this study are available from the corresponding author upon reasonable request.

## Supporting Information

Supporting Information 1 Search strategy and results.

Supporting Information 2 Full texts screened and reasons for exclusion.

Supporting Information 3 Data extraction table.

Supporting Information 4 Quality assessment.

Supporting Information 5 Odds ratios for CGM use.
