# Supplementary material for: Equity of Continuous Glucose Monitoring in Children and Young People With Type 1 Diabetes: A Systematic Review
Source: Pediatr Diabetes. 2025 Jun 3;2025:8875203. doi: 10.1155/pedi/8875203 (PMC12151618; doi:10.1155/pedi/8875203)
Supplement: Supporting Information 1 — Search strategy and results. [file 8875203.f1.docx]

Supplementary Material 1.

Search strategy and results.

| Medline via Ovid | 2352 |
| --- | --- |
| Embase via Ovid | 1382 |
| Web of Science Core Collection | 611 |
| Total | 4345 |
| Total Deduplicated | 3693 |

Searches run 4^th^ July 2023

Limited to publication date 2000 to present.

Health inequalities filter adapted from Prady *et al.,* 2018.

Prady, S.L., Uphoff, E.P., Power, M. *et al.* Development and validation of a search filter to identify equity-focused studies: reducing the number needed to screen. *BMC Med Res Methodol* **18**, 106 (2018). <https://doi.org/10.1186/s12874-018-0567-x>

Continuous glucose monitoring filter adapted from Elbalshy *et al.,* 2022.

Elbalshy M, Haszard J, Smith H, *et al.* Effect of divergent continuous glucose monitoring technologies on glycaemic control in type 1 diabetes mellitus: A systematic review and meta‐analysis of randomised controlled trials. *Diabetic Medicine* 2022; **39**. DOI:10.1111/dme.14854.

# Medline

Ovid MEDLINE(R) and Epub Ahead of Print, In-Process, In-Data-Review & Other Non-Indexed Citations, Daily and Versions <1946 to July 03, 2023>

1. Residence Characteristics/ or Environment design/ or exp Marital status/ or neighbo?rhood*.mp. or residential environment*.mp. or rural*.mp. or inner?city.mp. or housing instability.mp. or housing insecurity.mp. or housing strain.mp. or housing security.mp. or mortgage problems.mp. or foreclosure.mp. or eviction*.mp. or housing loss.mp. or home repossession*.mp. or home ownership.mp. or (repossess* adj3 hous*).mp. or (repossess* adj3 propert*).mp. or mortgage delinquency.mp. or mortgage arrears.mp. or mortgage debt*.mp. or overcrowding.mp. or (living adj1 (outside or inside or near* or adjacent)).mp. or (household adj2 size).mp. or (marital status or marriage status).mp. or (widow* or cohabit* or divorce* or single parent* or live* alone).mp.

357944

1. Cultural Deprivation/ or Acculturation/ or Culture/ or Cross-Cultural Comparison/ or Cultural Characteristics/ or Cultural Diversity/ or Language/ or "Transients and Migrants"/ or exp "Emigrants and Immigrants"/ or Minority groups/ or Minority health/ or Prejudice/ or Racism/ or Xenophobia/ or Social Discrimination/ or exp Race Relations/ or exp Ethnic Groups/ or exp Continental Population Groups/ or Refugees/ or minorit*.mp. or migration background.mp. or racial.mp. or racism.mp. or ethnology.mp. or race.mp. or ethnic*.mp. or non?English.mp. or language other than.mp. or latino*.mp. or latina*.mp. or hispanic*.mp. or whites.mp. or caucasian*.mp. or non?white.mp. or Torres Strait Islander.mp. or aboriginal.mp. or native american.mp. or inuit.mp. or eskimo.mp. or first nation*.mp. or indigenous.mp. or english as a second language.mp. or foreign language.mp.

827763

1. Occupations/ or Unemployment/ or occupations.mp. or unemployment.mp.
    66468
2. exp Gender Identity/ or Women's Health/ or gender differences.mp. or (sex disparit* or sex difference?).mp. or gender identity.mp. or sex role.mp. or wom#n* role?.mp. or m#n* role?.mp. or gender* role?.mp. or servicewomen.mp. or Sex factors/
    395141
3. exp Educational status/ or Education/ or Schooling.mp. or educational status.mp. or (education* adj2 level?).mp. or ((higher or better or worse or less) adj educated).mp. or ((higher or better or worse or less) adj level? of education).mp.
    151483
4. Religion/ or religi*.mp.
    72559
5. Social determinants of Health/ or Psychosocial Deprivation/ or Sociological Factors/ or Working Poor/ or Hierarchy, Social/ or disparit*.mp. or inequalit*.mp. or inequit*.mp. or equity.mp. or deprivation.mp. or gini.mp. or concentration index.mp. or Socioeconomic Factors/ or Social Welfare/ or exp Social Class/ or exp Poverty/ or Income/ or Social class*.mp. or social determinants.mp. or social status.mp. or social position.mp. or social background.mp. or social circumstance*.mp. or socio-economic.mp. or socioeconomic.mp. or sociodemographic.mp. or sociodemographic.mp. or SES.mp. or disadvantaged.mp. or impoverished.mp. or poverty.mp. or economic level.mp. or assets index.mp. or income*.mp.
    825756
6. Social Stigma/ or social capital/ or Social Control, Informal/ or exp Social Support/ or exp Social Environment/ or Trust/ or Social conditions/ or Social isolation/ or Social marginalization/ or Anomie/ or social participation/ or social exclusion.mp. or (social adj (capital or cohes* or organis* or organiz*)).mp. or (community adj3 (cohes* or participa*)).mp. or ((neighbourhood or neighborhood) adj cohes*).mp. or social relationships.mp. or social network*.mp. or collective efficacy.mp. or civil society.mp. or informal social control.mp. or neighbo*rhood disorder.mp. or social disorgani?ation.mp. or anomie.mp. or social support.mp. or social participation.mp. or trust.mp. or emotional support.mp. or psychosocial support.mp. or community capital.mp. or neighbo*rhood cohesion.mp. or social influence.mp. or (soci*context* or soci*-context*).mp.

339877

1. Health Status Disparities/ or Health Services Accessibility/ or Health Equity/ or health*care disparit*.mp. or health care disparit*.mp. or health status disparit*.mp. or health disparit*.mp. or health inequalit*.mp. or health inequit*.mp. or medically underserved.mp.
    151088
2. exp Health Status Disparities/ or exp Healthcare Disparities/ or exp Health Services Accessibility/ or exp health equity/ or (Medically underserved or disparit* or ((inequal* or equity or equal*) adj3 access*) or "private health insurance" or private insurance or public insurance or government insurance or commercial insurance or insurance status).ti,ab,kw,kf. or ((health* or health*care) adj3 (disparit* or equal* or unequal or inequalit* or equit* or inequit* or access* or inaccess* or gap* or gradient* or variation* or disadvantage*)).ti,ab,kw,kf. or ((racial or ethnic* or gender* or sex) and (minorit* or differ* or disparit*)).ti,ab,kw,kf. or ((high* or low*) adj2 (educat* or income*)).ti,ab,kw,kf.
    953181
3. exp Diabetes Mellitus, Type 1/ or (type 1 diabet* or type i diabet* or insulin dependent diabet* or t1d or iddm or t1dm or diabetes mellitus type 1 or diabetes mellitus type i or diabetes type 1 or diabetes type i or dm1 or juvenile onset diabet* or juvenile diabet*).ti,ab,kw,kf. or ((diabet* adj3 (type 1 or type i or insulin dependent or juvenile onset or juvenile or child* or p?ed* or youth or adolescen*)) or dm1 or t1d or t1dm or iddm).ti,ab,kw,kf.
    140787
4. exp Blood Glucose Self-monitoring/ or exp glycated hemoglobin/ or (continuous glucose monitor* or flash or continuous subcutaneous glucose or glucosesensor or glucose sensor or cgm or rtcgm or fgm or icgm or iscgm or diabetes technology or sensorguided or sensor guided or sensor augmented or sensoraugmented or sap).ti,ab,kw,kf.
    95799
5. or/1-10
    2734777
6. 13 and 11 and 12
    2862
7. limit 14 to yr="2000-Current"
    2352

# EMBASE

Embase <1974 to 2023 July 03>

1. demography/ or environmental planning/ or marriage/ or divorce/ or cohabitation/ or widow/ or exp "single (marital status)"/ or neighbo?rhood*.mp. or residential environment*.mp. or rural*.mp. or inner?city.mp. or housing instability.mp. or housing insecurity.mp. or housing strain.mp. or housing security.mp. or mortgage problems.mp. or foreclosure.mp. or eviction*.mp. or housing loss.mp. or home repossession*.mp. or home ownership.mp. or (repossess* adj3 hous*).mp. or (repossess* adj3 propert*).mp. or mortgage delinquency.mp. or mortgage arrears.mp. or mortgage debt*.mp. or overcrowding.mp. or (living adj1 (outside or inside or near* or adjacent)).mp. or (household adj2 size).mp. or (marital status or marriage status).mp. or (widow* or cohabit* or divorce* or single parent* or live* alone).mp.
    750457
2. exp cultural deprivation/ or cultural factor/ or cultural anthropology/ or cultural diversity/ or exp migrant/ or minority group/ or minority health/ or prejudice/ or exp social discrimination/ or exp race relation/ or exp ethnic group/ or exp ancestry group/ or exp refugee/ or minorit*.mp. or migration background.mp. or racial.mp. or racism.mp. or ethnology.mp. or race.mp. or ethnic*.mp. or non?English.mp. or language other than.mp. or latino*.mp. or latina*.mp. or hispanic*.mp. or whites.mp. or caucasian*.mp. or non?white.mp. or Torres Strait Islander.mp. or aboriginal.mp. or native american.mp. or inuit.mp. or eskimo.mp. or first nation*.mp. or indigenous.mp. or english as a second language.mp. or foreign language.mp.
    1215807
3. exp employment status/ or job characteristics/ or occupations.mp. or unemployment.mp.

71701

1. exp gender identity/ or women's health/ or sex difference/ or (sex disparit* or sex difference?).mp. or gender identity.mp. or sex role.mp. or wom#n* role?.mp. or m#n* role?.mp. or gender* role?.mp. or servicewomen.mp.
    515267
2. exp educational status/ or schooling.mp. or educational status.mp. or (education* adj2 level?).mp. or ((higher or better or worse or less) adj educated).mp. or ((higher or better or worse or less) adj level? of education).mp.
    212590
3. religion/ or religi*.mp.
    102662
4. "social determinants of health"/ or social aspect/ or working poor/ or exp social hierarchy/ or socioeconomics/ or disparit*.mp. or inequalit*.mp. or inequit*.mp. or equity.mp. or deprivation.mp. or gini.mp. or concentration index.mp. or social welfare/ or social class/ or poverty/ or social status/ or social background/ or social class*.mp. or social determinants.mp. or social status.mp. or social position.mp.
    791221
5. (social background or social circumstance* or socio-economic or socioeconomic or sociodemographic or socio-demographic or SES or disadvantaged or impoverished or poverty or economic level or assets index or income*).mp.
    622227
6. exp social isolation/ or social capital/ or social stigma/ or social support/ or social environment/ or trust/ or exp social exclusion/ or anomie/ or social participation/ or social exclusion.mp. or (social adj (capital or cohes* or organis* or organiz*)).mp. or (community adj3 (cohes* or participa*)).mp. or ((neighbourhood or neighborhood) adj cohes*).mp. or social relationships.mp. or social network*.mp. or collective efficacy.mp. or civil society.mp. or informal social control.mp. or neighbo*rhood disorder.mp. or ocial disorgani?ation.mp. or anomie.mp. or social support.mp. or social participation.mp. or trust.mp. or emotional support.mp. or psychosocial support.mp. or community capital.mp. or neighbo*rhood cohesion.mp. or social influence.mp. or (soci*context* or soci*-context*).mp.
    395555
7. health disparity/ or health equity/ or health care access/ or health*care disparit*.mp. or health care disparit*.mp. or health status disparit*.mp. or health disparit*.mp. or health inequalit*.mp. or health inequit*.mp. or medically underserved.mp.
    165388
8. (association* between or (positively associated or negatively associated) or differed by or (were high* amongst or were low* amongst) or (inverse relationship with or inversely associated with or inversely related to) or reverse association or differentially affects or evidence of a link between or (significantly adj3 likelihood of) or protective factors for or (differ* adj2 according to) or (inverse adj2 gradient) or (positive adj2 gradient) or (negative adj2 gradient) or (trends were adj3 across) or (related to adj3 variable*) or (differences were adj3 explained by) or (significant among or no# significant among)).mp.
    1237803
9. exp Health Status Disparities/ or exp Healthcare Disparities/ or exp Health Services Accessibility/ or exp health equity/ or (Medically underserved or disparit* or ((inequal* or equity or equal*) adj3 access*) or "private health insurance" or private insurance or public insurance or government insurance or commercial insurance or insurance status).ti,ab,kw,kf. or ((health* or health*care) adj3 (disparit* or equal* or unequal or inequalit* or equit* or inequit* or access* or inaccess* or gap* or gradient* or variation* or disadvantage*)).ti,ab,kw,kf. or ((racial or ethnic* or gender* or sex) and (minorit* or differ* or disparit*)).ti,ab,kw,kf. or ((high* or low*) adj2 (educat* or income*)).ti,ab,kw,kf.
    1305171
10. or/1-12
     4706076
11. exp *insulin dependent diabetes mellitus/ or (type 1 diabet* or type i diabet* or insulin dependent diabet* or t1d or iddm or t1dm or diabetes mellitus type 1 or diabetes mellitus type i or diabetes type 1 or diabetes type i or dm1 or juvenile onset diabet* or juvenile diabet*).ti,ab. or ((diabet* adj3 (type 1 or type i or insulin dependent or juvenile onset or juvenile or child* or p?ed* or youth or adolescen*)) or dm1 or t1d or t1dm or iddm).ti,ab.
     183329
12. exp *blood glucose monitoring/ or exp *glycosylated hemoglobin/ or (continuous glucose monitor* or flash or continuous subcutaneous glucose or glucosesensor or glucose sensor or cgm or rtcgm or fgm or icgm or iscgm or diabetes technology or sensorguided or sensor guided or sensor augmented or sensoraugmented or sap).ti,ab. 92036
13. 13 and 14 and 15
     3189
14. limit 16 to yr="2000 -Current"
     2947
15. limit 17 to embase
     1382

# Web of Science

# Web of Science Search Strategy (v0.1)

# Database: Web of Science Core Collection

# Entitlements:

- WOS.IC: 1993 to 2023
- WOS.CCR: 1985 to 2023
- WOS.SCI: 1900 to 2023
- WOS.AHCI: 1975 to 2023
- WOS.BHCI: 2008 to 2023
- WOS.BSCI: 2008 to 2023
- WOS.ESCI: 2018 to 2023
- WOS.ISTP: 1990 to 2023
- WOS.SSCI: 1956 to 2023
- WOS.ISSHP: 1990 to 2023

# Searches:

1: TS= (neighbo?rhood* or "residential environment* " or inner?city or "housing instability " or "housing insecurity " or "housing strain " or "housing security " or "mortgage problems " or foreclosure or eviction* or "housing loss " or "home repossession* " or "home ownership " or (repossess* near/3 hous*) or (repossess* near/3 propert*) or "mortgage delinquency " or "mortgage arrears " or "mortgage debt* " or overcrowding or (living near/1 (outside or inside or near* or adjacent) ) or (household near/2 size) or ("marital status " or "marriage status ") or (widow* or cohabit* or divorce* or single parent* or live* alone) )

Date Run: Tue Jul 04 2023 11:22:02 GMT+0100 (British Summer Time) Results: 237730

2: TS= (neighbo?rhood* or "residential environment* " or inner?city or "housing instability " or "housing insecurity " or "housing strain " or "housing security " or "mortgage problems " or foreclosure or eviction* or "housing loss " or "home repossession* " or "home ownership " or (repossess* near/3 hous*) or (repossess* near/3 propert*) or "mortgage delinquency " or "mortgage arrears " or "mortgage debt* " or overcrowding or (living near/1 (outside or inside or near* or adjacent) ) or (household near/2 size) or ("marital status " or "marriage status ") or (widow* or cohabit* or divorce* or single parent* or live* alone) )
 Date Run: Tue Jul 04 2023 11:22:09 GMT+0100 (British Summer Time)

Results: 237730

3: TS=(minorit* or "migration background" or racial or racism or ethnology or race or ethnic* or non?English or "language other than" or latino* or latina* or hispanic* or whites or caucasian* or non?white or "Torres Strait Islander " or aboriginal or "native american " or inuit or eskimo or "first nation* " or indigenous or "english as a second language " or "foreign language" or "cultural deprivation" or acculturation or "cultural diversity" or transient* or migrant* or emigra* or immigra* or "minority group*" or prejudic* or "cultural bias" or discriminat* or refugee*)
 Date Run: Tue Jul 04 2023 11:22:12 GMT+0100 (British Summer Time)

Results: 2977759

4: TS= (occupation* or unemploy*)

Date Run: Tue Jul 04 2023 11:22:17 GMT+0100 (British Summer Time)

Results: 339126

5: TS= ("sex disparit*" or "sex difference*" or "gender identity" or "sex role" or "sex factor*"or

"wom?n* role*" or "m?n* role*" or "gender* role*" or servicewomen or "gender difference*")

Date Run: Tue Jul 04 2023 11:22:22 GMT+0100 (British Summer Time)

Results: 227141

6: TS=(Schooling or "educational status" or (education* near/2 level*) or ((higher or better or worse or less) near/1 educated) or ((higher or better or worse or less) near/1 level* of education))

Date Run: Tue Jul 04 2023 11:22:26 GMT+0100 (British Summer Time)

Results: 918264

7: TS= (religi*)

Date Run: Tue Jul 04 2023 11:22:30 GMT+0100 (British Summer Time)

Results: 256708

8: TS=(disparit* or inequalit* or inequit* or equity or deprivation or gini or "concentration index" or "Social class*" or "social determinant*" or "social status" or "social position" or "social background" or "social circumstance*" or socio-economic or socioeconomic or sociodemographic or sociodemographic or SES or disadvantaged or impoverished or poverty or "economic level" or "assets index" or income*)

Date Run: Tue Jul 04 2023 11:22:34 GMT+0100 (British Summer Time)

Results: 1559813

9: TS= ("social exclusion" or (social near/1 (capital or cohes* or organis* or organiz*) ) or

(community near/3 (cohes* or participa*) ) or ((neighbourhood or neighborhood) near/1 cohes*) or "social relationship*" or "social network*" or "collective efficacy" or "civil society" or "informal social control" or "neighbo*rhood disorder" or "social disorgani?ation" or anomie or "social support" or "social participation" or trust or "emotional suppor"t or "psychosocial support" or "community capital" or "neighbo*rhood cohesion" or "social influence" or (soci*context* or soci*-context*) )

Date Run: Tue Jul 04 2023 11:22:38 GMT+0100 (British Summer Time)

Results: 592311

10: TS= ("health*care disparit*" or "health status disparit*" or "health disparit*" or "health inequalit*" or "health inequit*" or "medically underserved" or "health services accessibilty")

Date Run: Tue Jul 04 2023 11:22:42 GMT+0100 (British Summer Time)

Results: 43377

11: TS=("Medically underserved" or disparit* or ((inequal* or equity or equal*) near/3 access*) or "private health insurance" or "private insurance" or "public insurance" or "government insurance" or "commercial insurance" or "insurance status" or ((health* or health*care) near/3 (disparit* or equal* or unequal or inequalit* or equit* or inequit* or access* or inaccess* or gap* or gradient* or variation* or disadvantage*)) or ((racial or ethnic* or gender* or sex) and (minorit* or differ* or disparit*)) or ((high* or low*) near/2 (educat* or income*)))

Date Run: Tue Jul 04 2023 11:25:40 GMT+0100 (British Summer Time)

Results: 1366312

12: #11 OR #10 OR #9 OR #8 OR #7 OR #6 OR #5 OR #4 OR #3 OR #2 OR #1

Date Run: Tue Jul 04 2023 11:25:47 GMT+0100 (British Summer Time)

Results: 6631834

13: TS=("type 1 diabet*" or "type i diabet*" or "insulin dependent diabet*" or t1d or iddm or t1dm or "diabetes mellitus type 1" or "diabetes mellitus type I" or "diabetes type 1" or "diabetes type I" or dm1 or "juvenile onset diabet*" or "juvenile diabet*" or ((diabet*) near/3 ("type 1" or "type I" or "insulin dependent" or "juvenile onset" or juvenile or child* or ped* or paed* or youth or adolescen*)) or dm1 or t1d or t1dm or iddm)

Date Run: Tue Jul 04 2023 11:28:16 GMT+0100 (British Summer Time)

Results: 126228

14: TS= ("continuous glucose monitor*" or flash or "continuous subcutaneous glucose" or glucosesensor or "glucose sensor" or cgm or rtcgm or fgm or icgm or iscgm or "diabetes technology" or sensorguided or "sensor guided" or "sensor augmented" or sensoraugmented or sap)

Date Run: Tue Jul 04 2023 11:29:01 GMT+0100 (British Summer Time)

Results: 165624

15: #12 AND #13 AND #14

Date Run: Tue Jul 04 2023 11:29:09 GMT+0100 (British Summer Time)

Results: 618

16: #12 AND #13 AND #14 Timespan: 2000-01-01 to 2023-12-31

Date Run: Tue Jul 04 2023 11:29:36 GMT+0100 (British Summer Time)

Results: 611
