# Supplementary material for: Equity of Continuous Glucose Monitoring in Children and Young People With Type 1 Diabetes: A Systematic Review
Source: Pediatr Diabetes. 2025 Jun 3;2025:8875203. doi: 10.1155/pedi/8875203 (PMC12151618; doi:10.1155/pedi/8875203)
Supplement: Supporting Information 4 — Quality assessment. [file 8875203.f4.docx]

**Supplementary Material 4 – Quality assessment**

Please note that all quality assessments were performed with regards to our specific outcomes of interest, in line with the Newcastle-Ottawa Scale guidance.^1^ For many studies included in this review, inequalities in CGM use or HbA1c of CGM users were not the primary outcome of interest. Some therefore scored relatively poorly in this quality assessment, principally in the ‘comparability’ and ‘statistical test’ domains, despite robust methodology in general.

**Key**

| **Very good** | 9-10 |
| --- | --- |
| **Good** | 7-8 |
| **Satisfactory** | 5-6 |
| **Unsatisfactory** | 0-4 |

Study scoring system adapted from Nyawo *et al.*, 2022.^2^

**Table 1. Cross-sectional studies assessing CGM use as outcome of interest.**

| Study | **1) Representativeness of sample (★)** | **2) Sample size (★)** | **3) Non-respondents (★)** | **4) Ascertainment of exposure (risk factor) (★★)** | **1) Comparability (★★)** | **1) Assessment of outcome (★★)** | **2) Statistical test (★)** | **Total (/10)** |
| --- | --- | --- | --- | --- | --- | --- | --- | --- |
| Addala et al., 2021^3^ | ★ |  |  | ★ | ★★ | ★★ | ★ | **7** |
| DeSalvo et al., 2018^4^ | ★ |  |  | ★★ |  | ★★ |  | **5** |
| Foster et al., 2019^5^ | ★ |  |  | ★★ |  | ★★ |  | **5** |
| Wong et al., 2014^6^ | ★ |  | ★ | ★ |  | ★ |  | **4** |
| Sawyer et al., 2022^7^ | ★ |  | ★ | ★★ |  | ★★ | ★ | **7** |
| Alonso et al., 2023^8^ |  |  | ★ | ★★ |  | ★★ |  | **5** |
| Lee et al., 2021^9^ | ★ |  | ★ | ★★ |  | ★★ |  | **6** |
| Lipman et al., 2020^10^ | ★ |  |  | ★★ |  | ★★ | ★ | **6** |
| Lai et al., 2021^11^ | ★ |  |  | ★★ | ★★ | ★★ | ★ | **8** |
| Choudary et al., 2022^12^ | ★ |  |  | ★★ |  | ★★ |  | **5** |
| Sheikh et al., 2018^13^ | ★ |  |  | ★★ | ★★ | ★★ | ★ | **8** |
| Auzanneau et al., 2018^14^ | ★ |  |  | ★★ | ★★ | ★★ | ★ | **8** |
| Auzanneau et al., 2021^15^ | ★ |  |  | ★★ | ★★ | ★★ | ★ | **8** |
| Kordonouri et al., 2019^16^ | ★ |  |  |  |  |  |  | **1** |
| Stanley et al., 2023^17^ | ★ |  | ★ | ★★ |  | ★★ |  | **6** |
| Bratke et al., 2021^18^ | ★ |  | ★ | ★★ |  | ★★ | ★ | **7** |
| Burnside et al., 2023^19^ | ★ |  | ★ | ★★ | ★★ | ★★ | ★ | **9** |
| Šumník et al., 2021^20^ | ★ |  |  | ★★ |  | ★★ | ★ | **6** |
| Delagrange et al., 2020^21^ | ★ | ★ | ★ | ★ |  | ★★ |  | **6** |
| NPDA 2021/2022^22^ | ★ |  | ★ | ★★ |  | ★★ |  | **6** |
| NPDA 2020/2021^23^ | ★ |  | ★ | ★★ |  | ★★ |  | **6** |
| NPDA 2019/2020^24^ | ★ |  | ★ | ★★ |  | ★★ |  | **6** |
| NPDA 2018/2019^25^ | ★ |  | ★ | ★★ |  | ★★ |  | **6** |
| NPDA 2017/2018 ^26^ | ★ |  | ★ | ★★ |  | ★★ |  | **6** |

**Table 2. Cohort studies assessing CGM use as outcome of interest.**

| Study | **1) Representativeness of exposed cohort (★)** | **2) Selection of the non exposed cohort (★)** | **3) Ascertainment of exposure (★)** | **4) Demonstration that outcome of interest was not present at start of study (★)** | **1) Comparability of cohorts on the basis of the design or analysis (★★)** | **1) Assessment of outcome (★)** | **2) Was follow-up long enough for outcomes to occur (★)** | **3) Adequacy of follow-up of cohorts (★)** | **Total (/9)** |
| --- | --- | --- | --- | --- | --- | --- | --- | --- | --- |
| Ravi et al., 2021^27^ | ★ | ★ | ★ |  |  | ★ |  |  | **4** |
| Tremblay et al., 2023^28^ | ★ | ★ | ★ | ★ |  | ★ | ★ |  | **6** |
| Ladd, 2022^29^ | ★ | ★ | ★ | ★ | ★★ | ★ | ★ | ★ | **9** |

**Table 3. Cross-sectional studies HbA1c of CGM users as outcome of interest.**

| Study | **1) Representativeness of sample (★)** | **2) Sample size (★)** | **3) Non-respondents (★)** | **4) Ascertainment of exposure (risk factor) (★★)** | **1) Comparability (★★)** | **1) Assessment of outcome (★★)** | **2) Statistical test (★)** | **Total (/10)** |
| --- | --- | --- | --- | --- | --- | --- | --- | --- |
| Lee et al., 2021^9^ | ★ |  | ★ | ★★ | ★★ | ★★ | ★ | **9** |
| Kordonouri et al., 2019^16^ | ★ |  |  |  |  |  |  | **1** |
| Burnside et al., 2023^19^ | ★ |  | ★ | ★★ | ★★ | ★★ | ★ | **9** |

**References**

1 Wells G, Shea B, O’Connell D, *et al.* The Newcastle-Ottawa Scale (NOS) for assessing the quality of nonrandomised studies in meta-analyses. Ottawa Hospital Research Institute. 2013.

2 Nyawo TA, Dludla P V., Mazibuko-Mbeje SE, *et al.* A systematic review exploring the significance of measuring epicardial fat thickness in correlation to B-type natriuretic peptide levels as prognostic and diagnostic markers in patients with or at risk of heart failure. *Heart Fail Rev* 2022; **27**: 665–75.

3 Addala A, Auzanneau M, Miller K, *et al.* A Decade of Disparities in Diabetes Technology Use and HbA1c in Pediatric Type 1 Diabetes: A Transatlantic Comparison. *Diabetes Care* 2021; **44**: 133–40.

4 DeSalvo DJ, Miller KM, Hermann JM, *et al.* Continuous glucose monitoring and glycemic control among youth with type 1 diabetes: International comparison from the T1D Exchange and DPV Initiative. *Pediatr Diabetes* 2018; **19**: 1271–5.

5 Foster NC, Beck RW, Miller KM, *et al.* State of Type 1 Diabetes Management and Outcomes from the T1D Exchange in 2016–2018. *Diabetes Technol Ther* 2019; **21**: 66–72.

6 Wong JC, Foster NC, Maahs DM, *et al.* Real-Time Continuous Glucose Monitoring Among Participants in the T1D Exchange Clinic Registry. *Diabetes Care* 2014; **37**: 2702–9.

7 Sawyer A, Sobczak M, Forlenza GP, Alonso GT. Glycemic Control in Relation to Technology Use in a Single-Center Cohort of Children with Type 1 Diabetes. *Diabetes Technol Ther* 2022; **24**: 409–15.

8 Alonso GT, Triolo TM, Akturk HK, *et al.* Increased Technology Use Associated With Lower A1C in a Large Pediatric Clinical Population. *Diabetes Care* 2023; **46**: 1218–22.

9 Lee JM, Rusnak A, Garrity A, *et al.* Feasibility of Electronic Health Record Assessment of 6 Pediatric Type 1 Diabetes Self-management Habits and Their Association With Glycemic Outcomes. *JAMA Netw Open* 2021; **4**: e2131278.

10 Lipman TH, Smith JA, Patil O, Willi SM, Hawkes CP. Racial disparities in treatment and outcomes of children with type 1 diabetes. *Pediatr Diabetes* 2021; **22**: 241–8.

11 Lai CW, Lipman TH, Willi SM, Hawkes CP. Racial and Ethnic Disparities in Rates of Continuous Glucose Monitor Initiation and Continued Use in Children With Type 1 Diabetes. *Diabetes Care* 2021; **44**: 255–7.

12 Choudhary A, Adhikari S, White PC. Impact of the COVID-19 pandemic on management of children and adolescents with Type 1 diabetes. *BMC Pediatr* 2022; **22**: 124.

13 Sheikh K, Bartz SK, Lyons SK, DeSalvo DJ. Diabetes Device Use and Glycemic Control among Youth with Type 1 Diabetes: A Single-Center, Cross-Sectional Study. *J Diabetes Res* 2018; **2018**: 1–6.

14 Auzanneau M, Lanzinger S, Bohn B, *et al.* Area Deprivation and Regional Disparities in Treatment and Outcome Quality of 29,284 Pediatric Patients With Type 1 Diabetes in Germany: A Cross-sectional Multicenter DPV Analysis. *Diabetes Care* 2018; **41**: 2517–25.

15 Auzanneau M, Rosenbauer J, Maier W, *et al.* Heterogeneity of Access to Diabetes Technology Depending on Area Deprivation and Demographics Between 2016 and 2019 in Germany. *J Diabetes Sci Technol* 2021; **15**: 1059–68.

16 Kordonouri O, Lange K, Biester T, *et al.* Determinants of glycaemic outcome in the current practice of care for young people up to 21 years old with type 1 diabetes under real‐life conditions. *Diabetic Medicine* 2020; **37**: 797–804.

17 Stanley JR, Clarke ABM, Shulman R, Mahmud FH. Mediating Effects of Technology-Based Therapy on the Relationship Between Socioeconomic Status and Glycemic Management in Pediatric Type 1 Diabetes. *Diabetes Technol Ther* 2023; **25**: 186–93.

18 Bratke H, Margeirsdottir HD, Assmus J, Njølstad PR, Skrivarhaug T. Does Current Diabetes Technology Improve Metabolic Control? A Cross-Sectional Study on the Use of Insulin Pumps and Continuous Glucose Monitoring Devices in a Nationwide Pediatric Population. *Diabetes Therapy* 2021; **12**: 2571–83.

19 Burnside MJ, Williman JA, Davies HM, *et al.* Inequity in access to continuous glucose monitoring and health outcomes in paediatric diabetes, a case for national continuous glucose monitoring funding: a cross-sectional population study of children with type 1 diabetes in New Zealand. *Lancet Reg Health West Pac* 2023; **31**: 100644.

20 Šumník Z, Pavlíková M, Pomahačová R, *et al.* Use of <scp>continuous glucose monitoring</scp> and its association with type 1 diabetes control in children over the first 3 years of reimbursement approval: Population data from the <scp>ČENDA</scp> registry. *Pediatr Diabetes* 2021; **22**: 439–47.

21 Delagrange M, Dalla‐Vale F, Salet R, *et al.* Impact of deprivation on glycaemic control in youth with type 1 diabetes in the southwestern region of France. *Pediatr Diabetes* 2021; **22**: 796–806.

22 Royal College of Paediatrics and Child Health. National Paediatric Diabetes Audit Annual Report 2021-22: Care Processes and Outcomes. London, 2023.

23 Royal College of Paediatrics and Child Health. National Paediatric Diabetes Audit Annual Report 2020-21: Care Processes and Outcomes. London, 2022.

24 Royal College of Paediatrics and Child Health. National Paediatric Diabetes Audit Annual Report 2019-20: Care Processes and Outcomes. London, 2021.

25 Royal College of Paediatrics and Child Health. National Paediatric Diabetes Audit 2018/19: Care Processes and Outcomes. London, 2020.

26 Royal College of Paediatrics and Child Health. National Paediatric Diabetes Audit 2017/18: Care Processes and Outcomes. London, 2019.

27 Ravi SJ, Coakley A, Vigers T, Pyle L, Forlenza GP, Alonso T. Pediatric Medicaid Patients With Type 1 Diabetes Benefit From Continuous Glucose Monitor Technology. *J Diabetes Sci Technol* 2021; **15**: 630–5.

28 Tremblay ES, Bernique A, Garvey K, Astley CM. A Retrospective Cohort Study of Racial/Ethnic and Socioeconomic Disparities in Initiation and Meaningful Use of Continuous Glucose Monitoring among Youth With Type 1 Diabetes. *J Diabetes Sci Technol* 2023; published online July 3. DOI:10.1177/19322968231183985.

29 Ladd J. Glycemic control and socioeconomic status in Canadian children with type 1 diabetes using continuous glucose monitoring: A retrospective cohort study. 2022; published online April 15.
